# Seasonality shapes gut microbiota composition in two sympatric sea urchins

**DOI:** 10.7717/peerj.20918

**Published:** 2026-03-05

**Authors:** Ruber Rodríguez-Barreras, Jaleniz Suárez-Pérez, Filipa Godoy-Vitorino

**Affiliations:** 1 Biology Dept., University of Puerto Rico at Bayamon, Bayamón, PR, United States of America; 2 School of Medicine, Microbiology and Immunology Dept, University of Puerto Rico Medical Sciences Campus, San Juan, PR, United States of America

**Keywords:** Gut microbiota, 16S rRNA sequencing, Sea urchins, Diadema, Echinometra, Sympatric species

## Abstract

**Background:**

The gut microbiota plays critical roles in digestion, immunity, and environmental adaptation in marine invertebrates. Its composition is shaped by both host traits and abiotic factors such as temperature and seasonality. In Caribbean reefs, sea urchins like *Diadema antillarum* and *Echinometra lucunter* are important grazers that regulate algal biomass and influence benthic community dynamics. This study used 16S rRNA gene sequencing to compare, for the first time, the gut microbiota of these two sympatric species across contrasting seasons.

**Methods:**

Adults of *D. antillarum* and *E. lucunter* were collected during summer and winter from three fringing reefs in Puerto Rico. Gut contents were extracted under sterile conditions, and bacterial DNA was extracted amplified for their 16S rRNA genes and sequenced. Microbial diversity and structure were assessed standard microbiota pipelines.

**Results:**

Microbial communities in both echinoids were dominated by Bacillota, Bacteroidota, Desulfobacterota, and Pseudomonadota. Core microbiota analysis revealed stable taxa across hosts and seasons, most notably *Propionigenium*, supporting host-driven selection mechanisms that maintain functional stability despite seasonal turnover. Core microbiome analyses revealed Bacillota, Bacteroidota, Desulfobacterota_G_459543, and Pseudomonadota as persistent phyla across seasons, and genera such as *Desulfotalea*, *Photobacterium*, and *Propionigenium* consistently present in both species. Alpha diversity was significantly higher in winter for both *D. antillarum* and *E. lucunter*, while species-level differences were not significant. Beta diversity analyses showed significant seasonal clustering, with no consistent segregation between species within seasons. Our findings demonstrate that shared environmental conditions, particularly seasonality, are the primary modulators of gut microbiota in sympatric sea urchins, while host selection preserves a conserved core community. This dual influence of environment and host highlights the ecological plasticity and resilience of sea urchin microbiotas under fluctuating reef conditions.

## Introduction

Sea urchins play a key ecological role in shallow benthic ecosystems, functioning as important grazers that regulate algal biomass and shape the dynamics of coral reef communities (Williams & Polunin, 2001; Bilodeau, Layman & Silman, 2021). Sea urchins promote coral and fish recruit survival by removing macroalgae and maintaining open substrate spaces, contributing to the resilience of coral reefs (Dang et al., 2020). Among the most common species in these environments are *Diadema antillarum* and *Echinometra lucunter*, both widely distributed along the coasts of Puerto Rico and throughout the Caribbean (Rodríguez-Barreras, Sabat & Calzada-Marrero, 2013). These two species share similar rocky substrate and shallow reef habitats but differ in aspects of their functional ecology, including feeding behavior, population density, and mobility. *D. antillarum* tends to form denser aggregations and is highly mobile, while *E. lucunter* is more dispersed and tends to shelter in crevices (Hendler et al., 1995). Since both species feed mainly on algae and epibenthic material, their intestines function as ecological filters, reflecting the microbial composition of the surrounding environment and their diet (Rodríguez-Barreras, Tosado-Rodríguez & Godoy-Vitorino, 2021).

The microbiome plays a critical role in the physiology, nutrition, development, and immunity of marine organisms, including echinoderms (Hentschel et al., 2012; Rizzo & Lo Giudice, 2018; Rathinam et al., 2024). Recent advances in high-throughput sequencing technologies and bioinformatic tools have revolutionized the study of microbial communities, enabling more precise and in-depth characterization of the microbiota associated with marine organisms (Hakim et al., 2019; Schwob et al., 2020; Rodríguez-Barreras et al., 2023). As in other marine invertebrates, the gut microbiota of sea urchins plays vital roles in digestion, metabolism, immune regulation, and pathogen defense (Carrier et al., 2021; Haditomo et al., 2021). Understanding the composition and variability of these symbiotic communities is therefore crucial, given their central involvement in host digestion, immunity, metabolism, and adaptation to environmental changes (Schoeler & Caesar, 2019; Zheng et al., 2024; Zhang et al., 2025). In recent decades, the use of molecular tools, particularly 16S rRNA gene sequencing, has enabled accurate characterization of bacterial communities in a wide range of marine invertebrates (Hakim et al., 2016; Godoy-Vitorino & Toledo-Hernandez, 2018; Astudillo-García et al., 2020). Although the microbiota of sea urchins had been understudied using advanced molecular techniques, recent studies have helped bridge this knowledge gap in this class of echinoderm, focusing on the characterization of intestinal and epibiotic microbiota, as well as the effects of disease, body size, and seasonal variation on the gut microbial communities (Rodríguez-Barreras, Tosado-Rodríguez & Godoy-Vitorino, 2021; Rodríguez-Barreras et al., 2023; Rodríguez-Barreras et al., 2024; Ruiz-Barrionuevo et al., 2024).

The microbiota structure is highly sensitive to environmental factors, such as temperature, salinity, and nutrient availability (Hou et al., 2017; Cornejo-Granados et al., 2018; Marangon et al., 2023). For instance, fluctuations in environmental variables such as temperature are known to influence microbial community structure in marine species (Gao et al., 2018; Ketchum et al., 2021; Rodríguez-Barreras et al., 2024). Recent evidence indicates that increases in seawater temperature may lead to greater variability in the host-associated microbial community. For example, a recent study on a species of the genus *Echinometra* reported that gut microbiota composition varies with environmental temperature, highlighting the importance of characterizing the spatial and temporal patterns of these microbial communities under varying conditions (Ketchum et al., 2021). The study also revealed that certain bacterial taxa, such as *Propionigenium* and members of the Vibrionaceae, respond consistently to seasonal changes in temperature.

Seasonality is a major selective force shaping host-associated microbiota, acting both through physiological changes and direct effects on microbial growth dynamics (Deng et al., 2022; Ketchum et al., 2021). During the summer months in the northern hemisphere, rising temperatures has been associated with reduction in microbial diversity (Brothers et al., 2018; Gutiérrez et al., 2018; Li et al., 2018; Ketchum et al., 2021). This pattern suggests that under elevated temperatures or thermal stress, the microbiota may become more variable and less diverse. Assessing whether these dynamics also occur in Caribbean echinoids that share the same habitat is essential for understanding the functional stability of these ecosystems. While seasonal variation in the gut microbiota has been documented in the sea urchin *Tripneustes ventricosus* (Rodríguez-Barreras et al., 2024), the influence of seasonality of two sympatric species, *D. antillarum* and *E. lucunter*, remains to be characterized.

One of the earliest studies on the microbiota of *D. antillarum* and *E. lucunter* examined the eggs of both species from either side of the Isthmus of Panama, showing that the host’s evolutionary history along with environmental factors shapes microbial communities (Carrier et al., 2021). In the Caribbean, our research group has been characterizing the microbiota of four sea urchin species advancing knowledge beyond previous studies that focused on single seasons or fewer species (Rodríguez-Barreras, Tosado-Rodríguez & Godoy-Vitorino, 2021; Rodríguez-Barreras et al., 2023; Rodríguez-Barreras et al., 2024). In this study we compare the seasonal variation of the gut microbiota in *D. antillarum* and *E. lucunter* collected from the same habitat, providing new insights into how host biology and environmental factors interact to shape these microbial communities. We hypothesized that seasonal variation would exert a stronger influence on gut microbial communities than species-specific differences, even among sea urchins inhabiting the same environment. To investigate this, we set three goals: (i) describe the gut microbiota of *Diadema antillarum* and *Echinometra lucunter* during the summer, (ii) compare their gut microbiota in summer between both sea urchin species, and (iii) examine how gut microbial communities change between summer and winter within each species.

## Materials & Methods

### Sample collection

We randomly collected six adults at three sites off the coast of Puerto Rico by site of each sea urchin species (*Diadema antillarum* and *Echinometra lucunter*) during August of 2019, for a total of 18 individuals of each species, and a grand total of 36 sea urchin samples. Sampling collection was approved by the Department of Natural and Environmental Resources of Puerto Rico (Permit number DRNA-2019-IC-003). Site collections were carried out on different days within the same month for each location to avoid potential mixing of individuals among sites. Additionally, we collected 1 L of seawater in a sterile glass container per site. Sea urchins were placed in separate containers filled with seawater and transported in an insulated foam cooler for subsequent processing.

### Environmental variables

Both species were collected in close proximity within the same habitat at three shallow-water (1–2 m depth) fringing reefs along the northeastern coast of Puerto Rico. Collection sites included Cerro Gordo in Vega Baja (CG; 18°29′05.81″N, 66°20′20.23″W), Isla de Cabra in Toa Baja (IC; 18°28′26.32″N, 66°08′18.82″W), and Punta Bandera in Luquillo (MA; 18°23′15.01″N, 65°43′11.26″W). Four abiotic parameters—salinity (ppt), water temperature (°C), dissolved oxygen, and pH—were measured at each site in February and August using a Pro2030 quality meter (Xylem Inc., Washington, DC). Each abiotic parameter was averaged from five replicate measurements, and comparative tests were conducted to determine potential temporal differences. According to Rodríguez-Barreras et al. (2024), temperature was the only environmental parameter that showed significant differences between February and August.

### Sample processing

Once in the laboratory, the sea urchins were subjected to a chemical procedure to induce euthanasia. This chemical procedure is commonly used in marine invertebrates (Arafa, Sadok & Abed, 2007; Wahltinez, Harms & Lewbart, 2025). The euthanization process was performed following the Institutional Animal Care and Use Committee (IACUC) protocol of the University of Puerto Rico Medical Sciences Campus (Permit number A-5301118). Each individual was placed in a 100 mL glass beaker containing seawater for at least 10 min to allow attachment to the surface. Sedation was then induced by adding 25 mL of a 20 mM magnesium chloride (MgCl_2_⋅6H_2_O) solution. Following sedation, sea urchins gradually detached from the beaker walls and were subsequently transferred to a metal tray, where they were exposed to ultra-low temperatures (−80°C) for 10 min prior to dissection. Each sea urchin body was then carefully opened by making an equatorial incision around the oral membrane using flame-sterilized scissors to avoid any damage to the digestive system during dissection (Whalen, 2008). The gut was then removed, opened, and the fecal pellets were transferred to a Petri dish using sterile tweezers. Fecal pellets were subsequently placed in two mL sterile microtubes and stored at −80°C until DNA extraction. This procedure focused on isolating the bacterial community associated with gut digesta, specifically excluding tissue-associated bacteria.

### DNA extraction, PCR amplification and sequencing

DNA was extracted from fecal pellets by using the QIAGEN PowerSoil™ Kit (QIAGEN LLC, Germantown, MD, USA), with few modifications to the manufacturer’s instructions which included: fecal pellet samples were homogenized using a PowerLyzer homogenizer for 2 min at room temperature at 3,000 rpm. During the elution step, 100 µL of sterile PCR-grade water preheated to 65°C was added to the spin column and incubated for 5 min before a final centrifugation. DNA concentrations were quantified using the Qubit^®^ dsDNA HS Assay Kit and Qubit^®^ Fluorometer (Thermo Fisher Scientific, Waltham, MA, USA), with values ranging from 5 to 100 ng/µL. A negative extraction (blank) control was processed alongside the samples to monitor background contamination.

DNA extracts were normalized to 4 nM for 16S rRNA library preparation. The V4 hypervariable region of the 16S rRNA gene was amplified using the universal primers 515F (5′GTGCCAGCMGCCGCGGTAA3′) and 806R (5′GGACTACHVGGGTWTCTAAT3′), following the Earth Microbiome Project protocol (Caporaso et al., 2012) and conditions described in Abarca et al. (2018). Amplicons were sequenced using the Illumina MiSeq platform with a 2  × 250 bp paired-end configuration (targeting the V4 region). The sequence data obtained for *D. antillarum* and *E. lucunter* collected in August 2019 (summer season) were submitted to Qiita (Gonzalez et al., 2018) under Bioproject ID-15707. The new summer sequencing data are now publicly available in the European Nucleotide Archive (ENA) under accession numbers PRJEB90128 (ERP173143). For comparative analyses, we used previously published winter-season (February 2019) gut microbiota data for the same species, which were submitted to Qiita under Bioproject ID 12668 (ENA projects PRJEB40117 and ERP123720); see Rodríguez-Barreras, Tosado-Rodríguez & Godoy-Vitorino, 2021 for details. Both the winter and summer samples were collected by us and processed and sequenced in the same laboratories. The winter data were already public when we analyzed the current datasets reported in this paper, and we have now submitted the newly sequenced summer datasets. All datasets for both species are publicly available through the European Nucleotide Archive.

### Bioinformatic analyses, data preprocessing, and statistical testing

Raw read preprocessing of the demultiplexed FASTQ files was performed within the Qiita platform using the split_libraries_fastq.py script (QIIME2, v1.9.1) with a Phred quality score offset of 33 and default parameters (Bolyen et al., 2019). Amplicon sequence variants (ASVs) were then generated using Deblur (2021.09), within Qiita, following data preprocessing steps that included trimming to 250 bp to standardize length prior to denoising. Taxonomic classification was performed using the Greengenes2 database as reference (McDonald et al., 2024). This was to maintain full compatibility with the Qiita processing pipeline and to ensure consistent taxonomic classification across both previously published winter data and newly generated summer samples. Greengenes2 provides a unified reference tree and stable taxonomy framework that is specifically optimized for cross-study integration within Qiita. Both datasets were therefore processed using identical pipelines, parameters, and database versions. The resulting feature tables and representative sequences were then merged at the QIIME2 feature table and representative sequence level, ensuring compatibility across seasons.

The first sequencing study deposited in Qiita, titled “Habitat conditions reflect compositional differences in microbiome among Caribbean Sea urchins” (Qiita ID: 12668), originally comprised 127 samples from N species collected during the winter season. For our current analysis, however, we focused exclusively on the gut microbiota data from both *E. lucunter* and *D. antillarum* sea urchin species, narrowing the dataset to 36 samples. The second sequenced study, titled “Temporal dynamics of gut microbiota in *E. lucunter* and *D. antillarum* Caribbean sea urchins” (Qiita ID: 15707), consisted of 18 samples collected during the summer season, of which 14 samples were retained. Together, these datasets provided 50 total samples across two species and two seasons.

The merged dataset (winter and summer) was subsequently divided into three analytical subsets to address the specific biological questions: (i) samples from both species collected during the summer and winter, (ii) E. lucunter samples collected across both seasons, and (iii) D. antillarum samples collected across both seasons. To ensure methodological consistency and enable valid comparisons, all three subsets were rarefied to a single sequencing depth of 1,670 reads, corresponding to the minimunm read depth across the merged feature table. Prior to rarefaction, the dataset was filtered to remove singleton features, chloroplast and mitochondrial sequences, rare eukaryotic sequences, and features unclassified at the phylum level, ensuring that downstream analyses were based only on features with at least phylum-level resolution. In addition, all ASVs detected in the negative extraction control (10 reads in total) were identified in the unrarefied table and removed from all samples prior to rarefaction and downstream analyses. The metadata variables considered were species (*D. antillarum* or *E. lucunter*), season (winter or summer), and the interaction term species_season, defined by four group combinations: black_winter, black_summer, red_winter, and red_summer.

### Alpha and beta diversity

We calculated and plotted alpha diversity using four metrics implemented in QIIME2. Faith’s phylogenetic diversity (Faith’s PD) to capture phylogenetic richness (Faith, 1992), and Pielou’s evenness to characterize community evenness (Pielou, 1966); these two metrics served as the primary alpha diversity measures, following recommendations to evaluate the two major dimensions of within-sample diversity. For completeness and to facilitate comparison with prior gut microbiome studies, we also calculate Chao-1 richness (Kuczynski et al., 2012), and Shannon diversity (Caporaso et al., 2010). All four indices were computed using the qiime diversity alpha and qiime diversity alpha-phylogenetic pipelines, and group differences were assessed with the qiime diversity alpha-group-significance command in QIIME2, which applies a non-parametric Kruskal-Wallis test (Bolyen et al., 2019). Beta diversity visualization was performed using Principal Coordinates Analysis (PCoA) based on Bray-Curtis dissimilarity, which represents compositional dissimilarities between microbial communities across samples in a reduced two-dimensional space. To incorporate phylogenetic relationships among taxa, we also calculated both unweighted and weighted Unique Fraction (UniFrac) distance matrices (Lozupone & Knight, 2005) within QIIME 2 and visualized them using PCoA. To evaluate temporal and species-specific variation in microbial composition, we assessed seasonal differences within each species and between seasons using permutational analysis of variance (PERMANOVA) and analysis of similarities (ANOSIM) (Anderson, 2001), applied to all beta diversity matrices (Bray-Curtis, unweighted UniFrac, and weighted UniFrac) *via* the qiime diversity beta-group-significance plugin in QIIME 2 (Bolyen et al., 2019; Martino et al., 2019). Statistical significance was determined using 999 permutations for each test. Boxplots were generated using ggplot2 (Wickham, 2009; R Core Team, 2025).

### Taxonomic profiles and biomarkers

Taxonomic composition at the phylum and genus levels was elucidated through taxa bar plots generated in RStudio (R Core Team, 2025). For visualization, the top 20 most abundant taxa were displayed at each taxonomic level (phylum and genus), across all comparisons, to improve clarity and interpretability. To further characterize the shared microbial community across samples, core feature plots were generated at genus levels using prevalence thresholds to identify taxa consistently present across varying proportions of individuals. To identify taxa that act as putative biomarkers by ranking microbial features based on their ability to distinguish between seasons and species, we applied Random Forest analysis (Breiman, 2001). For this we used MicrobiomeAnalyst (Dhariwal et al., 2017), which employs Mean Decrease Accuracy as its statistical ranking metric. Furthermore, multivariable associations between microbial taxa and metadata categories were assessed using MaAsLin2 (Mallick et al., 2021), a generalized linear modeling framework that accounts for fixed effects and multiple covariates, to identify significantly associated taxa at the phylum and genus levels. In these Maaslin2 models, “season” and “species” were included as the primary fixed effects of interest, while “qiita_study_id” was added as a fixed effect covariate to correct for potential batch effects introduced by sequencing runs. It is worth noting that the phylum Firmicutes, traditionally reported in intestinal microbiota studies, has been reclassified as Bacillota under the Genome Taxonomy Database (GTDB). For consistency with MaAsLin2 outputs and current GTDB standards, we report these taxa using the updated Bacillota nomenclature while acknowledging their historical classification as Firmicutes.

## Results

### Summary of reads and ASVs

A total of 50 samples were analyzed, showing an average of 37,044 ± 26,835 sequencing reads and 922.82 ± 522.10 amplicon sequence variants (ASVs) (Table 1). The summer samples (ID-15707) included 14 samples from both species, selected from an initial pool of 33 individuals after rarefaction (see Methods). The average number of reads was 65,489 ± 28,713 with 527.57 ± 280.50 amplicon sequence variants (ASVs). The sea urchin *Diadema antillarum* exhibited lower ASV richness (mean 508.57 ± 283.16 ASVs) compared to *Echinometra lucunter*, which had 546.57 ± 299.09 ASVs. In winter (ID-12668), a total of 36 samples were processed, showing an average of 25,982 ± 15,888 reads per sample and an ASV richness of 1,076.53 ± 515.37. During this season, *E. lucunter* showed the highest values for both sequencing depth (30,947 ± 16,389) and ASV richness (1,217.49 ± 519.66), compared to *D. antillarum* (*n* = 18), which averaged 21,017 ± 14,104 reads and 935.11 ± 484.19 ASVs.

**Table 1:** Number of samples, mean read counts (± SD), and amplicon sequence variant (ASV) richness (± SD) by species and season. Values are shown prior to rarefaction for the sea urchins *Diadema antillarum* and *Echinometra lucunter* sampled in summer (Qiita ID 12668) and winter (Qiita ID 15707). Mean values and standard deviations are reported across samples within each group.

	**Total count**	**Average** ± **SD of reads**	**Average** **± SD** ** of ASVs**
*Summer* (Qiita ID-15707)(ENA: PRJEB90128/ERP173143)	14	65,489.21 ± 28,713.90	527.57 ± 280.50
*Black*	7	71,235.14 ± 29,626.17	508.57 ± 283.16
*Red*	7	59,743.29 ± 28,838.23	546.57 ± 299.09
*Winter* (Qiita ID-12668)(ENA: PRJEB40117/ ERP123720)	36	25,982.72 ± 15,888.67	1,076.53 ± 515.37
*Black*	18	21,017.78 ± 14,104.40	935.11 ± 484.19
*Red*	18	30,947.67 ± 16,389.46	1,217.94 ± 519.66
Grand Total	50	37,044.54 ± 26,835.32	922.82± 522.10

### Spatio-temporal dynamic of the gut microbiota

**Figure 1:**
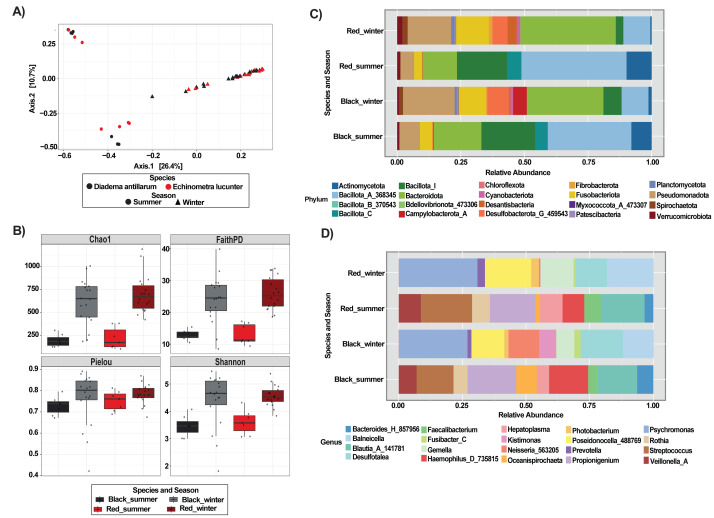
Gut microbiota structure and composition of sea urchins *Diadema antillarum* and *Echinometra lucunter*) across species and seasons (summer and winter). (A) PCoA analysis of Bray Curtis dissimilarities. (B) Alpha diversity indices (Chao1, Faith’s PD, Shannon, and Pielou). Taxonomic composition at the phylum (C) and genus level (D).

Principal Coordinates Analysis (PCoA), based on Bray-Curtis dissimilarities revealed a strong seasonal structuring of gut microbiota, rather than separation by host-species. Within each season, both sea urchin species largely overlap with no consistent species-level segregation (Fig. 1A). Analyses using unweighted and weighted UniFrac distances revealed clustering patterns consistent with those observed using Bray-Curtis dissimilarities (Fig. S1). Pairwise PERMANOVA and ANOSIM tests confirmed signifcant differences among most species-season combinations (*p* < 0.05), except for summer samples of *D. antillarum* and *E. lucunter*, which did not differ significantly under any distance metric (Bray-Curtis: ANOSIM *p* = 0.775, PERMANOVA *p* = 0.701; UniFrac: ANOSIM weighted *p* = 0.867, PERMANOVA weighted *p* = 0.888; Tables S1 and S2).

Alpha diversity (Faith’s PD, Chao1 richness, Shannon diversity, and Pielou’s evenness) was numerically higher in *E. lucunter* than in *D. antillarum* during summer (Fig. 1B), but this species comparison was not statistically significant (Faith’s PD, *p* = 0.949; Chao 1, *p* = 0.0.949; Pielou, *p* = 0.337; Shannon, *p* = 0.0.337; Table S3). In contrast, seasonal effects were pronounced within both species. *D. antillarum* showed significantly greater richness and diversity in winter compared to summer (Chao1, *p* = 0.000; Shannon, *p* = 0.011; Faith’s PD, *p* = 0.002). *E. lucunter* likewise exhibited higher diversity and phylogenetic richness in winter (Chao1, *p* = 0.0001; Shannon, *p* = 0.000; Faith’s PD, *p* = 0.000; Table S3). Pielou’s evenness did not differ significantly between seasons for either species (all *p* > 0.05). Species comparison within winter samples were also non-significant (black winter *vs* red winter: Chao1, *p* = 0.486; Shannon, *p* = 0.635; Faith’s PD, *p* = 0.486; Pielou’s evenness, *p* = 0.680; Table S3).

Taxonomic bar plots at both the phylum and genus levels revealed similar microbial compositions between sea urchin species, although some compositional shifts were evident (Figs. 1C & 1D). For example, Bacillota_A_368345 was the most abundant phylum *in E. lucunter*, followed by Bacillota_I and Bacteroidota. In contrast, *D. antillarum* showed a similar phylum profile but with Bacillota_I being slightly more dominant than in *E. lucunter* (Fig. 1C). On the other hand, the phylum Campylobaterota_A was detected exclusively in *D. antillarum* samples collected during winter. The relative abundance of bacterial genera showed clear seasonal shifts in both sea urchin species (Fig. 1D). In winter, both *E. lucunter* (Red_winter) and *D. antillarum* (Black_winter) displayed a dominance of a few genera, with *Psychromonas*, *Poisedonocella_488769*, and Desulfotalea being more prevalent. In contrast, summer samples from both species exhibited higher proportions of genera such as *Streptococcus*, *Propionigenium*, and Blautia_A_141781, along with a more even distribution of additional taxa (Fig. 1D).

**Figure 2:**
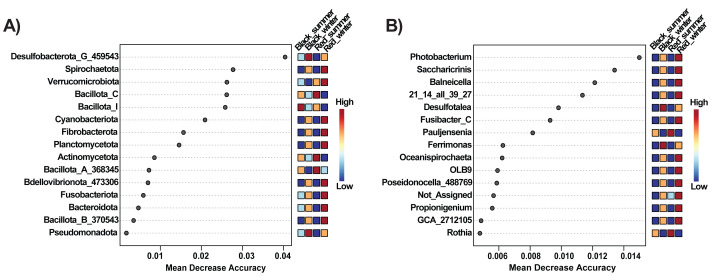
Important feature selection by Random Forest at the Phylum (A) and Genus (B) levels, showing species-seasonal variations of the bacterial taxa. The plots display the taxonomic features of importance and are ranked based on the mean decrease in classification accuracy when permuted. “High” and “Low” refer to a higher and lower value respectively, of the importance of that taxon in predicting the group.

Although both species share many microbial taxa, they maintain distinct microbial signatures that can be resolved by both taxonomic composition and predictive modeling. Random Forest analysis at the phylum level identified the bacterial groups with the greatest predictive importance for distinguishing *Diadema antillarum* from *Echinometra lucunter* across seasons. The phyla with the greatest predictive importance, as indicated by Mean Decrease Accuracy, were *Desulfobacterota_G_459543, Spirochaetota, Verrucomicrobiota, Bacillota_C,* and *Bacillota_I* (Fig. 2A). At the genus level, the top predictors were *Photobacterium, Saccharicrinis, Balneicella, 21_14_all_39_27, Desulfotalea,* and *Fusibacter_C* (Fig. 2B).

### Seasonal differences in *Echinometra lucunter*

**Figure 3:**
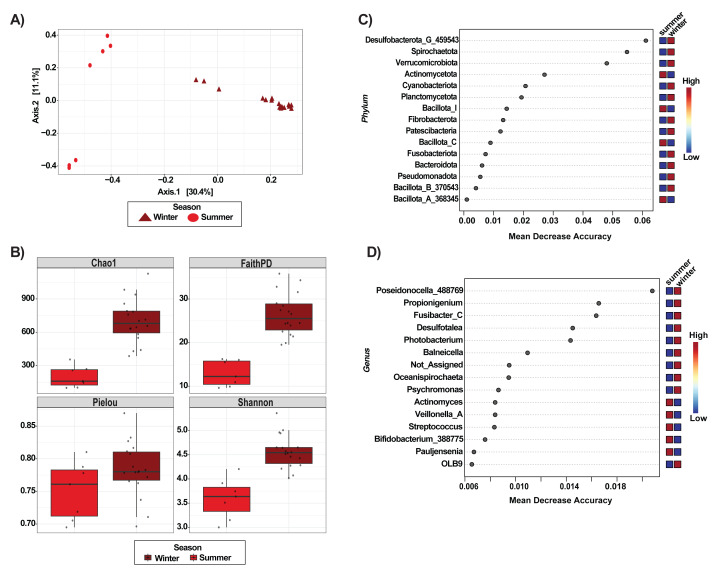
Seasonal variation in the gut microbiota of the sea urchin *Echinometra lucunter* across seasons. (A) Principal Coordinates Analysis (PCoA) based on Bray-Curtis dissimilarities show a clear separation between summer and winter samples. (B) Alpha diversity metrics (Chao1, Faith’s PD, Shannon, and Pielou) comparing summer and winter gut microbiota. Random Forest importance plots for phylum-level (C) and genus-level (D) taxa.

Seasonal changes in the gut microbiota of *E. lucunter* were found between summer and winter (Fig. 3). The PCoA analysis showed a clear separation of communities, with winter samples clustering distinctly from summer samples (Fig. 3A). Both PERMANOVA and ANOSIM confirmed that these seasonal differences were statistically significant (*p* = 0.001 for both tests; Table S4). Consistent with these results, PCoAs based on unweighted and weighted UniFrac distances also found seasonal separation between samples (Fig. S2), with significance further confirmed by ANOSIM and PERMANOVA (*p* = 0.001 for both distance metrics; Table S5). Alpha diversity analyses revealed that E. lucunter exhibited significantly higher richness and diversity during winter compared to summer (Chao1, *p* = 0.000; Shannon, *p* = 0.000; Faith’s PD, *p* = 0.000; Fig. 3B; Table S6). In contrast, evenness did not differ significantly between seasons (Pielou’s evenness, *p* = 0.102). These findings indicate that the gut microbiota of *E. lucunter* was more complex and diverse during winter.

**Figure 4:**
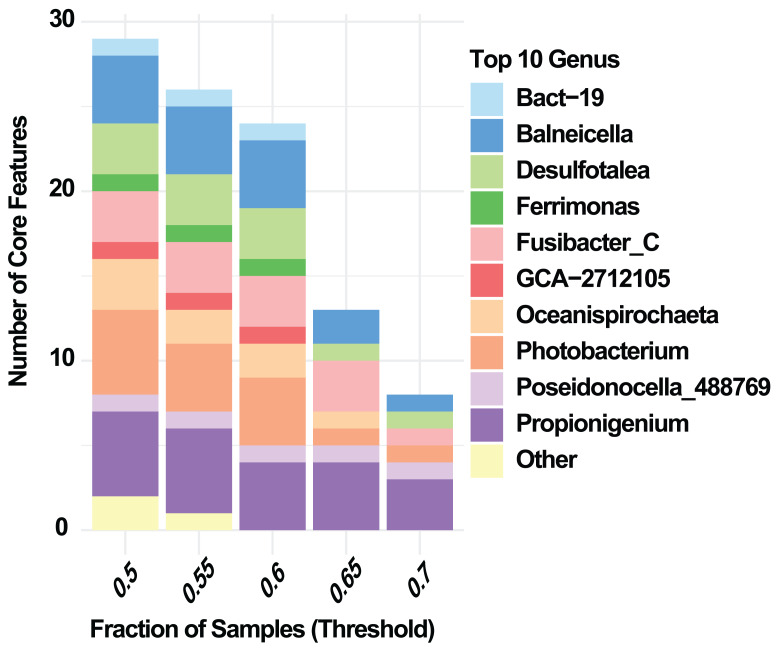
Genus-level core microbiome of the sea urchin *Echinometra lucunter* across prevalence thresholds. Number of core features at the genus level, limited to the ten most abundant genera, across prevalence thresholds (0.5–0.7). Core features were defined as taxa present in at least the specified fraction of samples.

At the phylum level, Random Forest analysis identified the taxa with the highest predictive importance for distinguishing summer and winter samples of *E. lucunter* (Fig. 3C). The phyla contributing most strongly to classification, based on Mean Decrease Accuracy, were *Desulfobacterota_G_459543, Spirochaetota, Verrucomicrobiot.* Other phyla, including *Actinomycetota, Cyanobacteriota*, and *Planctomycetota* showed lower but still detectable contributions (Fig. 3C). At the genus level, the analysis revealed that *Poseidonocella_488769* was the most important taxon for distinguishing between summer and winter samples. Other genera, including *Propionigenium, Fusibacter_C, Desulfotalea,* and *Photobacterium*, contributed to the classification with considerably lower importance (Fig. 3D). Core microbiome analysis at the genus level revealed a consistent set of shared taxa across both seasons , including *Balneicella, Desulfotalea, Fusibacter_C, Oceanispirochaeta*, *Photobacterium, and Propionigenium* (Fig. 4).

MaAsLin2 analysis revealed significant seasonal shifts in the gut microbiota of *E. lucunter*, with all significant assosciations at both phylum and genus levels enriched in winter samples. At the phylum level, the winter-enriched taxa included *Spirochaetota*, *Desulfobacterota_G_459543*, *Bacillota_C, Planctomycetota, Fibrobacterota, Cyanobacteriota, Actinomycetota, Bacillota_I, Pseudomonadota, and Patescibacteria* (Fig. S3). No phyla were significantly enriched in summer. At the genus level, all significantly associated taxa met the FDR threshold, with the majority enriched in winter samples. Including *Streptococcus*, *Photobacterium*, *Desulfotalea*, *Balneicella*, *Propionigenium*, *Poseidonocella_488769*, *Fusibacter_C*, *Oceanispirochaeta*, *Saccharicrinis*, and *Actinomyces* (Fig. S4). Together this results indicate a strong seasonal restructuring of the E. lucunter gut microbiota, driven by the markedly higher abundance of multiple phyla and genera during the winter season.

### Seasonal differences in *Diadema atillarum*

**Figure 5:**
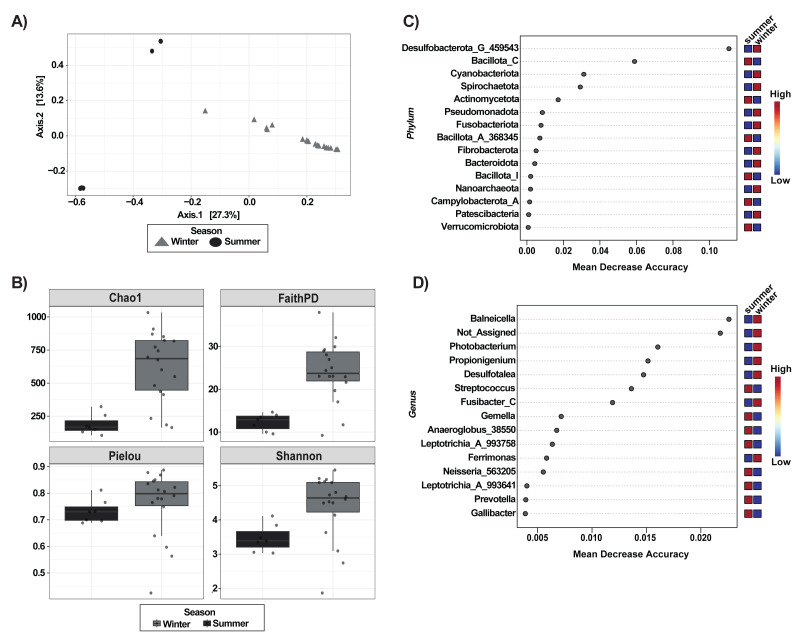
Seasonal variation in the gut microbiota of the sea urchin *Diadema antillarum* across seasons. (A) Principal Coordinates Analysis (PCoA) based on Bray-Curtis dissimilarities show a clear separation between summer and winter samples. (B) Alpha diversity metrics (Chao1, Faith’s PD, Shannon, Pielou). Random Forest importance plots for phylum-level (C) and genus-level (D) taxa.

Seasonal changes in the gut microbiota of *D. antillarum* were detected during the study (Fig. 5). The PCoA analysis showed a differences between summer and winter microbial communities, with summer samples clustered more tightly, while winter samples were more dispersed (Fig. 5A). Both PERMANOVA and ANOSIM tests indicated also significant seasonal differences (*p* = 0.001 for each test; Table S7). Consistent with these results, UniFrac-based analyses demonstrated strong phylogenetic shifts between seasons, with both unweighted and weighted UniFrac distances showing significant differences (ANOSIM and PERMANOVA, *p* = 0.001 for all; Fig. S5, Table S8). Alpha diversity analyses revealed that D. antillarum exhibited significantly higher richness and diversity in winter compared to summer (Chao1, *p* = 0.000; Shannon, *p* = 0.011; Faith’s PD, *p* = 0.001; Fig. 5B; Table S9). Evenness did not differ significantly between seasons (Pielou’s evenness, *p* = 0.102).

**Figure 6:**
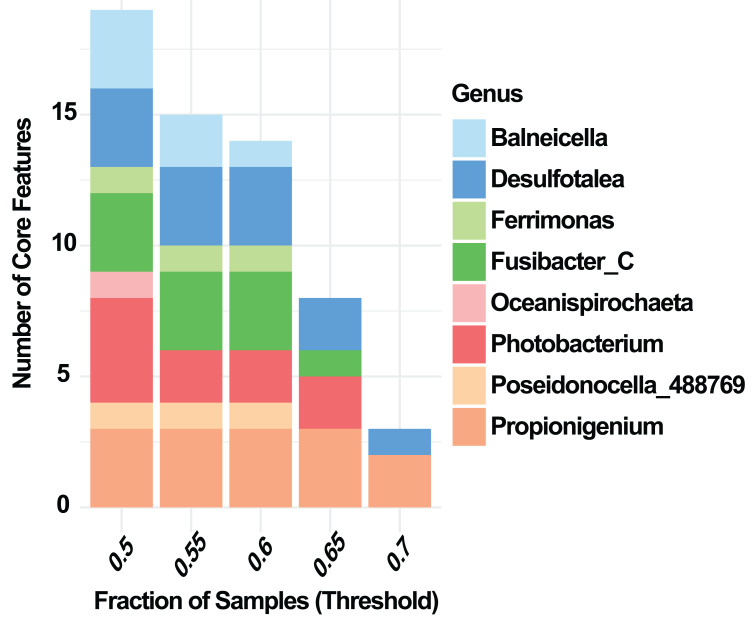
Genus-level core microbiome of the sea urchin *Diadema antillarum* across prevalence thresholds. Number of core features identified at the genus level across increasing prevalence thresholds (0.5–0.7). Only genera meeting the specified fraction of sample presence were retained as core members.

Random Forest analysis at the phylum level identified *Desulfobacterota_G_459543, followed by Bacillota_C, Cyanobacteriota, Spirochaetota, and Actinomycetota (*Fig. 5C*).* At the genus level, the taxa with the greatest predictive importance were *Balneicella, a not assigned genera, Photobacterium, Propionigenium, and Desulfotalea* (Fig. 5D). Core microbiome analysis at the genus level revealed a consistent set of taxa shared across summer and winter samples. Genera that persisted across increasing prevalence thresholds Balneicella, Desulfotalea, Ferrimonas, Fusibacter_C, Oceanispirochaeta, Photobacterium, Poseidonocella_488769, and Propionigenium (Fig. 6).

MaAsLin2 analysis identified significant seasonal differences in the gut microbiota of Diadema antillarum, with all significant associations enriched in winter. At the phylum level, winter-enriched taxa included *Bacillota_C, Desulfobacterota_G_459543, Planctomycetota, Cyanobacteriota, Spirochaetota, Actinomycetota, Fibrobacterota, Bdellovibrionota_473306, Nanoarchaeota,* and *Pseudomonadota* (Fig. S6). In the genus level, most genera were enriched in winter, including *Balneicella, Streptococcus, Fusibacter_C, Desulfotalea, Propionigenium, Photobacterium, Veillonella_A, Gemella, Actinomyces*, and *Poseidonocella_488769* (Fig. S7). These results indicate that seasonal restructuring of the gut microbiota in *D. antillarum* is largely driven by the increased prevalence of multiple bacterial genera during winter, while a few taxa associated with gut fermentation processes dominate in summer.

## Discussion

### Host microbiota in co-occurring species

A relevant finding of this study is that the sea urchins *Diadema antillarum* and *Echinometra lucunter* showed parallel seasonal pattern in their gut microbiota, despite belonging to different genera. Both species exhibited overlapping community compositions within seasons, with a consistent separation between summer and winter rather than between hosts. This indicates that seasonality, more than host identity, acts as the primary structuring factor. The winter season was characterized by the enrichment of several bacterial groups (*e.g.*, *Desulfobacterota_G_459543*, *Spirochaetota*, *Planctomycetota*; genera such as *Fusibacter_C*, *Balneicella*, and *Photobacterium*) in both species, while summer was associated with the enrichment of phyla such as *Bacillota_C*, *Actinomycetota*, and *Bacillota_I*, and genera such as *Streptococcus* and *Veillonella_A*.

Similarities in gut microbiota between bopth sea urchins is consistent with their sympatric distribution and ecological roles (Rodríguez-Barreras et al., 2015; Rodríguez-Barreras et al., 2016). Despite taxonomic differences, both sea urchins fulfill similar ecological roles by controlling macroalgal communities (Hendler et al., 1995), which suggests a similarity in their feeding sources. In fact, previous studies have reported similarities in both the internal and external microbiota of these two species, and the white sea urchin *Tripneustes ventricosus* has also shown microbial resemblance with *D. antillarum* and *E. lucunter* (Rodríguez-Barreras, Tosado-Rodríguez & Godoy-Vitorino, 2021; Rodríguez-Barreras et al., 2024). Although *T. ventricosus* is typically associated with seagrass beds, the species occasionally migrates to the forereef zone inhabited by *D. antillarum* and *E. lucunter* (Moses & Bonem, 2001). This finding suggests that shared environmental conditions (temperature, food availability, and habitat structure) act as common modulators of gut microbiota (Schmitt et al., 2012; Akter et al., 2023; Rodríguez-Barreras et al., 2024).

This evidence supports the existence of a coherent microbial community response among sympatric species that share a similar physical and trophic environment. A similar pattern among sympatric species has been observed in other marine invertebrates (Schmitt et al., 2012; Akter et al., 2023). Studies on marine sponges have reported that different species cohabiting in the same environment can harbor highly similar microbial communities, suggesting that the shared environment plays a significant role in microbiota structuring (Schmitt et al., 2012). A recent study also documented similarities in the intestinal microbial communities of cohabiting marine bivalves, *Crassostrea gigas* and *Mytilus galloprovincialis* (Akter et al., 2023). The gut bacterial communities of both molluss shared large numbers of common bacterial taxa and were influenced by both host species and the shared environment, suggesting that a common habitat can lead to microbiota similarities across different species living in the same setting (Akter et al., 2023).

### Possible effect of diet in host-microbiota

Dietary differences between *Diadema antillarum* and *Echinometra lucunter* in Puerto Rico have been previously documented using DNA-metabarcoding (Rodríguez-Barreras et al., 2020). In that study, both species behaved as generalist omnivores dominated by macroalgae, but showed clear quantitative contrasts in the composition of their food resources. For example, *E. lucunter* exhibited a higher proportion of macroalgae and a relatively greater contribution of metazoans, whereas *D. antillarum* displayed a more diversified dietary spectrum characterized by higher relative abundances of protists and fungi (Rodríguez-Barreras et al., 2020). However, since the site × species interaction was not significant, it is observed that the dietary patterns of each species are comparable across the sites analyzed in Puerto Rico.

*Echinometra lucunter*, widely distributed from the southern United States to Brazil, is mainly herbivorous but shows trophic plasticity, consuming filamentous and calcified macroalgae, cyanobacteria, sponges, mollusks, and crustaceans depending on resource availability (Hendler et al., 1995; Tavares et al., 2020; Barrios & Reyes, 2008; Reyes-Luján et al., 2015; Lodeiros & García, 2004; Cobb & Lawrence, 2005). Similarly, *Diadema antillarum*, a key herbivore for reef health, feeds primarily on calcareous and filamentous algae (*Halimeda*, *Dictyota*, *Laurencia*, *Ulva*) but also exhibits omnivory, with records of sponges, bryozoans, copepods, and even coral fragments in its diet (Carpenter, 1981; Hughes, 1989; Rodríguez-Barreras et al., 2015; Herrera-López et al., 2004; Karlson, 1983). External factors such as sargassum influxes can further alter its feeding habits and microbiome (Cabanillas-Terán et al., 2019). However, the scarcity of studies characterizing the microbiota of macroalgae in the Caribbean limits the ability to distinguish diet-derived microorganisms from resident gut taxa, highlighting the need for integrated analyses of algal and echinoid microbiomes to clarify trophic interactions and their functional roles in reef ecosystems.

Studies in the Caribbean have shown that macroalgal microbiomes are diverse and spatio-temporally variable, with *Ulva lactuca* along the Colombian coast dominated by Proteobacteria, Bacteroidetes, and Cyanobacteria (Comba González et al., 2021), distinct microbial shifts detected among stranded, seawater, and stored *Sargassum* in the Windward Islands (Hervé et al., 2021), and holopelagic *Sargassum* from the Great Atlantic Sargassum Belt revealing dynamic community changes across oceanic and coastal sites in Mexico and Florida (Mendonça et al., 2024). Together, these studies highlight the complexity and functional roles of algal microbiomes, shaped by environmental and ecological factors that influence marine trophic interactions.

The characterization of host microbiota linked to consumed items is challenged by the facultative and temporally variable diets of sympatric echinoids, which shift between herbivory and omnivory depending on resource availability, and by the scarcity of detailed studies on macroalgal microbiomes in the region. This limits direct comparisons between algal- and urchin-associated communities and complicates distinguishing diet-derived microorganisms from resident gut taxa.

### Seasonality as a modulator of the gut microbiota

Across seasons, some bacterial phyla remained consistently present in the gut microbiota, suggesting temporal stability at higher taxonomic levels, as also observed in other Caribbean sea urchins (Rodríguez-Barreras, Tosado-Rodríguez & Godoy-Vitorino, 2021; Rodríguez-Barreras et al., 2023). Despite environmental fluctuations, sea urchins appear to maintain characteristic microbial associations through host-driven selection, providing functional resilience for digestive and metabolic processes. A central example is *Propionigenium*, repeatedly identified as a core genus across species (Schink & Pfennig, 1982; Hakim et al., 2019; Rodríguez-Barreras, Tosado-Rodríguez & Godoy-Vitorino, 2021; Rodríguez-Barreras et al., 2024; Ruiz-Barrionuevo et al., 2024), known for propionate production and associated with host health, whose stable presence across taxa and environments suggests evolutionary adaptation and host regulation (Ketchum et al., 2021).

Most bacterial groups identified in this study showed higher relative abundance in winter, indicating ecological sensitivity to Caribbean temperature variation, consistent with seasonal shifts reported in *Tripneustes ventricosus* (Rodríguez-Barreras et al., 2024) and other marine invertebrates where temperature shapes gut microbial structure (Brockington & Clarke, 2001; Zhu et al., 2024). For instance, microbial diversity also increased in winter in Pacific oysters (*Crassostrea gigas*) and Mediterranean mussels (*Mytilus galloprovincialis*) (Akter et al., 2023). In our study, fermentative phyla such as Desulfobacterota_G_459543, Planctomycetota, Spirochaetota, and Fibrobacterota dominated in winter, whereas Bacillota (formerly Firmicutes) and Actinomycetota were more abundant in summer, possibly reflecting adaptation to warmer conditions or diets richer in simple carbohydrates and fresh macroalgae.

For taxonomic consistency, we used the GTDB classification, where Firmicutes has been reclassified as Bacillota, with Bacillota_C and Bacillota_I representing distinct subclades. The observed increase in Bacillota_C and Bacillota_I during summer contrasts with previous findings in the sea urchin *Tripneustes ventricosus*, where Firmicutes was reported as the most abundant clade in winter (Rodríguez-Barreras et al., 2024), likely reflecting interspecific differences in physiology or diet.

It is important to note that direct cross-comparisons among all samples were limited by differences in rarefaction depth across subsets, although within-group analyses remained robust. Potential batch effects from sequencing runs were controlled by including “qiita_study_id” as a covariate in MaAsLin2 models, minimizing their influence on the seasonal patterns reported.

### Ecological implications and evolutionary considerations

The sensitivity of the gut microbiota to seasonal changes has direct implications for the functional ecology of *D. antillarum* and *E. lucunter*, both key herbivores in rocky and reef ecosystems that regulate algal biomass and recycle organic matter (McClanahan & Muthiga, 2001; Williams & Polunin, 2001). Shifts in gut microbial communities may affect digestive efficiency and, consequently, the ability to control macroalgal growth (Park et al., 2023). This functional link between microbiota composition and digestion has long been recognized in echinoderms, where non-parasitic microbial symbioses provide essential enzymatic capabilities that the host lacks, particularly for the breakdown of algal polysaccharides (De Ridder & Foret, 2001). Recent evidence has strengthened this perspective. For example, an study showed that restructuring of gut microbiota in sea urchins directly influences digestive enzyme activity and nutrient assimilation (Koch, Wohlrab & Saborowski, 2025), while other demonstrated that bacterial consortia associated with different algal diets contribute to host nutritional flexibility through nitrogen fixation and metabolic complementation (Bengtsson et al., 2025).

Microbial patterns can act as indicators of host health and environmental stress. During the 2022 mass mortality of *D. antillarum* in Puerto Rico, significant microbial shifts were documented, including the loss of taxa such as *Propionigenium* and *Photobacterium* (Hewson et al., 2023; Ruiz-Barrionuevo et al., 2024). The disappearance of *Propionigenium*, a fermenter of simple carbohydrates (Sun & Xu, 2021), has been linked to physiological stress, highlighting the value of microbiome analyses for detecting early dysbiosis. In the context of climate change, such imbalances may reflect immune weakness or loss of essential metabolic functions, whereas a diverse and stable microbiota could enhance resilience against environmental pressures and pathogens.

The results presented here support the view that the microbiota of marine invertebrates is not a passive component but a coevolving unit with its host (Carrier & Reitzel, 2018; Schuh et al., 2020). Indeed, future studies could analyze the animals’ gut metagenomes to assess genome-level diversity and putative functions. In addition, experiments involving the gut manipulation of gnotobiotic animals could help clarify the specific activities of particular microbiome components in the animals’ physiology. The plasticity and specificity observed in the bacterial communities of *D. antillarum* and *E. lucunter* reflect ecological selection processes that may influence long-term adaptation and tolerance to climate change. Certain taxa may provide benefits beyond digestion, including thermoregulation, immune modulation, and detoxification of bioactive compounds (Ho et al., 2016; Rodríguez-Barreras et al., 2024). These symbiotic associations, shaped by both environmental cues and host physiology, may thus represent a pathway for adaptive modulation of diet and resilience under extreme or fluctuating conditions.

### Study limitations

This study has some limitations that should be acknowledged. First, part of the data analyzed here (the winter dataset) had been generated previously and has been incorporated into this study to enable seasonal comparisons. Although all samples were collected by the same research group, extracted using identical protocols, and sequenced in the same facility, the two datasets were processed at different time points (August 2019 for the winter samples and December 2024 for the summer samples). Such temporal separation in sequencing runs may introduce batch effects due to variations in reagent lots, instrument performance, or other run-specific factors. To mitigate this, both datasets were processed in Qiita using identical parameters (DEBLUR 2021.09), trimmed to 250 bp, taxonomically classified using Greengenes2, and merged at the QIIME2 feature-table and sequence level. Additionally, the variable qiita_study_id (12668 and 15707) was explicitly included as a fixed-effect covariate in the MaAsLin2 analysis to account for potential sequencing run effects. Second, rarefaction to a minimum depth of 1,670 reads across samples necessarily limits the detection of low-abundance taxa. Although higher sequencing depths (*e.g.*, ≥10,000 reads per sample) are often desirable for fine-scale taxonomic resolution, previous studies of marine invertebrate gut microbiota indicate that dominant community patterns and seasonal signals are robust even at lower sequencing depths when analyses focus on overall community structure rather than rare taxa. Nonetheless, we acknowledge that diet–host–microbiota relationships should be made cautiously given the low sequencing depth. Lastly, integrated analyses of macroalgal and echinoid microbiomes are therefore needed to clarify these relationships and to determine which microbes represent transient dietary inputs *versus* stable components of the sea urchin gut. Despite these limitations, the integration of new summer data with previously generated winter data provides valuable insight into the seasonal dynamics of gut microbiota in Caribbean sea urchins and contributes to a broader understanding of how environmental factors influence host–microbiome interactions.

## Conclusions

This study demonstrates that the sympatric sea urchins *Diadema antillarum* and *Echinometra lucunter*, despite belonging to different genera, share similar gut microbiota and exhibit parallel responses to seasonal changes. The strong similarity in microbial composition and diversity patterns suggests that shared environmental factors in reef habitats, particularly habitat structure and diet, play a crucial role in shaping their gut communities. Seasonal restructuring -although limited comparisons due to batch methodlogical limiations -was most evident in the reduced diversity observed during summer, while the persistence of taxa such as *Propionigenium* across both species and seasons points to the existence of a stable core microbiota likely maintained by host-driven selection. This core associations may contribute to digestive efficiency, immune modulation, and resilience to environmental fluctuations. Overall, these findings highlight the dual influence of environmental filtering and host regulation in structuring sea urchin microbiomes. Comparative analyses across sympatric and allopatric populations, as well as among size classes, will be essential to further disentangle the roles of ecological specialization and host physiology in driving microbiome convergence or divergence.

##  Supplemental Information

10.7717/peerj.20918/supp-1 Supplemental Information 1 Supplementary Tables

10.7717/peerj.20918/supp-2 Supplemental Information 2 Principal Coordinates Analysis (PCoA) plots of gut microbiota in the sea urchins *Diadema antillarum* and *Echinometra lucunter* across summer and winter seasons based on (A) unweighted UniFrac and (B) weighted UniFrac distance metrics

10.7717/peerj.20918/supp-3 Supplemental Information 3 Principal Coordinates Analysis (PCoA) plots gut microbiota in the sea urchin *Echinometra lucunter* during the summer and winter season, based on (A) unweighted UniFrac and (B) weighted UniFrac distance metrics

10.7717/peerj.20918/supp-4 Supplemental Information 4 Differential abundance analysis across the summer (*n* = 7) and winter (*n* = 18) seasons in the sea urchin *Echinometra lucunter* at the phylum level using MaAsLin2

10.7717/peerj.20918/supp-5 Supplemental Information 5 Differential abundance analysis across the summer (*n* = 7) and winter (*n* = 18) seasons in the sea urchin *Echinometra lucunter* at the genus level using MaAsLin2

10.7717/peerj.20918/supp-6 Supplemental Information 6 Principal Coordinates Analysis (PCoA) plots gut microbiota in the sea urchin *Diadema antillarum* during the summer and winter season, based on (A) unweighted UniFrac and (B) weighted UniFrac distance metrics

10.7717/peerj.20918/supp-7 Supplemental Information 7 Differential abundance analysis across the summer (*n* = 7) and winter (*n* = 18) seasons in the sea urchin *Diadema antillarum* at the phylum level using MaAsLin2

10.7717/peerj.20918/supp-8 Supplemental Information 8 Differential abundance analysis across the summer (*n* = 7) and winter (*n* = 18) seasons in the sea urchin *Diadema antillarum* at the genus level using MaAsLin2

## Additional Information and Declarations

### Data Availability

The following information was supplied regarding data availability: The winter sequence data is available at NCBI / European Nucleotide Archive: PRJEB40117 ERP123720. The summer sequence data is available at NCBI/European Nucleotide Archive: PRJEB90128 (ERP173143).
