# Supplementary material for: Seasonality shapes gut microbiota composition in two sympatric sea urchins
Source: PeerJ. 2026 Mar 5;14:e20918. doi: 10.7717/peerj.20918 (PMC12967421; doi:10.7717/peerj.20918)
Supplement: Supplemental Information 1 [file peerj-14-20918-s001.docx]

| **Supplementary Table 1.** Pairwise comparisons of gut microbiota beta diversity between the sea urchins Diadema antillarum and Echinometra lucunter and seasons (N = 50) using ANOSIM and PERMANOVA tests based on Bray–Curtis dissimilarities. Values represent p-values for each pairwise comparison, with significant results (p < 0.05) shown in bold. | | | | | |
| --- | --- | --- | --- | --- | --- |
| **Seasons and Species**  **(N=50)** |  | **Experimental Group 1** | **Experimental Group 2** | **ANOSIM**  ***p*-value** | **PERMANOVA**  ***p*-value** |
|  | *D. antillarum*  _summer | *D. antillarum*  _winter | 25 | **0.001** | **0.001** |
|  |  | *E. lucunter* _summer | 14 | 0.775 | 0.701 |
|  |  | *E. lucunter* _winter | 25 | **0.001** | **0.001** |
|  | *D. antillarum* _winter | *E. lucunter* _summer | 25 | **0.001** | **0.001** |
|  |  | *E. lucunter* _winter | 36 | **0.001** | **0.002** |
|  | *E. lucunter*_  summer | *E. lucunter* _winter | 25 | **0.001** | **0.001** |

| **Supplementary Table 2.** Pairwise beta diversity comparisons between the sea urchins Diadema antillarum and Echinometra lucunter across summer and winter (N = 50). Community structure differences were tested using ANOSIM and PERMANOVA based on both unweighted and weighted UniFrac distance metrics. Sample sizes and corresponding p-values are reported, with significant results (p < 0.05) shown in bold. | | | | | | |
| --- | --- | --- | --- | --- | --- | --- |
| Group 1 | Group 2 | Sample size | **ANOSIM P-value** | | **PERMANOVA P-value** | |
|  |  |  | Unifrac Unweighted | Unifrac  Weighted | Unifrac Unweighted | Unifrac Weighted |
| *D. antillarum*  _summer | *D. antillarum*  _winter | 25 | **0.001** | **0.001** | **0.001** | **0.001** |
|  | *E. lucunter* _summer | 14 | 0.979 | 0.867 | 0.954 | 0.888 |
|  | *E. lucunter* _winter | 25 | **0.001** | **0.001** | **0.001** | **0.001** |
| *D. antillarum*  _winter | *E. lucunter* _summer | 25 | **0.001** | **0.001** | **0.001** | **0.001** |
|  | *E. lucunter* _winter | 36 | **0.002** | **0.050** | **0.002** | **0.032** |
| *E. lucunter* _summer | *E. lucunter* _winter | 25 | **0.001** | **0.001** | **0.001** | **0.001** |

| **Supplementary Table 3.** Pairwise comparisons of alpha diversity between sea urchin species and seasons (N= 50) based on Chao1 and Faith PD for richness and Shannon and Pielou’s for eveness diversity indices. Values represent p-values from Kruskal–Wallis tests for each pairwise comparison, with significant results (p < 0.05) shown in bold. | | | | | | | |
| --- | --- | --- | --- | --- | --- | --- | --- |
| **Seasons and Species**  **(N=50)** |  | **Experimental Group 1** | **Experimental Group 2** | **Chao1**  ***p*-value** | **Shannon**  ***p*-value** | **Faith PD**  ***p*-value** | **Pielou’s**  ***p*-value** |
|  | *D. antillarum*  _summer | *D. antillarum*  _winter | 25 | **0.000** | **0.011** | **0.002** | 0.079 |
|  |  | *E. lucunter* _summer | 14 | 0.949 | 0.337 | 0.949 | 0.337 |
|  |  | *E. lucunter* _winter | 25 | **0.000** | **0.000** | **0.000** | **0.013** |
|  | *D. antillarum*  _winter | *E. lucunter* _summer | 25 | **0.000** | **0.015** | **0.001** | 0.250 |
|  |  | *E. lucunter* _winter | 36 | 0.486 | 0.635 | 0.486 | 0.680 |
|  | *E. lucunter* _summer | *E. lucunter* _winter | 25 | **0.000** | **0.000** | **0.000** | 0.130 |

| **Supplementary Table 4.**  Beta diversity contrasts for *E*chinometra *lucunter* (red, summer N = 7; winter N = 18; total N = 25). Seasonal differences were examined with ANOSIM and PERMANOVA based on Bray–Curtis dissimilarities. *P*-values are shown, with significant results in bold. | | | | | |
| --- | --- | --- | --- | --- | --- |
|  |  | **Experimental Group 1** | **Experimental Group 2** | **ANOSIM**  ***p*-value** | **PERMANOVA**  ***p*-value** |
| **Red species per season**  **(N=25)** | Summer (N=7) | Winter (N=18) | 25 | **0.001** | **0.001** |

| **Supplementary Table 5.** Seasonal (summer vs. winter) beta diversity comparison within the sea urchin Echinometra lucunter (N = 25; summer N = 7, winter N = 18). Statistical significance was assessed with ANOSIM and PERMANOVA using both weighted and unweighted UniFrac distance matrices. Significant p-values (< 0.05) are highlighted in bold. | | | | | | |
| --- | --- | --- | --- | --- | --- | --- |
| Group 1 | Group 2 | Sample size | **ANOSIM P-value** | | **PERMANOVA P-value** | |
|  |  |  | Unifrac Unweighted | Unifrac Weighted | Unifrac Unweighted | Unifrac Weighted |
| Summer | Winter | 25 | **0.001** | **0.001** | **0.001** | **0.001** |

| **Supplementary Table 6.**  Alpha diversity comparison of the sea urchin Echinometra lucunter (red) between summer (N = 7) and winter (N = 18) seasons (N = 25 total). Richness (Chao1 and Faith PD) and diversity/evenness (Shannon and Pielou) were evaluated using Kruskal–Wallis tests.  *P*-values are shown, with significant results in bold. | | | | | | | |
| --- | --- | --- | --- | --- | --- | --- | --- |
|  |  | **Experimental Group 1** | **Experimental Group 2** | **Chao1**  ***p*-value** | **Shannon**  ***p*-value** | **Faith PD**  ***p*-value** | **Pielou’s**  ***p*-value** |
| **Red species per season**  **(N=25)** | Summer (N=7) | Winter (N=18) | 25 | **0.000** | **0.000** | **0.000** | 0.102 |

| **Supplementary Table 7.**  Seasonal beta diversity analysis of the sea urchin Diadema antillarum (black, summer N = 7; winter N = 18; total N = 25). Community differences were tested with ANOSIM and PERMANOVA using Bray–Curtis distances.  *P*-values are shown, with significant results in bold. | | | | | |
| --- | --- | --- | --- | --- | --- |
|  |  | **Experimental Group 1** | **Experimental Group 2** | **ANOSIM**  ***p*-value** | **PERMANOVA**  ***p*-value** |
| **Black species per season**  **(N=25)** | Summer (N=7) | Winter (N=18) | 25 | **0.001** | **0.001** |

| **Supplementary Table 8.** Seasonal (summer vs. winter) beta diversity comparison within the sea urchin Diadema antillarum (N = 25; summer N = 7, winter N = 18). Analyses were performed with ANOSIM and PERMANOVA based on unweighted and weighted UniFrac distances. Reported values correspond to p-values, with significant results indicated in bold. | | | | | | |
| --- | --- | --- | --- | --- | --- | --- |
| Group 1 | Group 2 | Sample size | **ANOSIM P-value** | | **PERMANOVA P-value** | |
|  |  |  | Unifrac Unweighted | Unifrac Weighted | Unifrac Unweighted | Unifrac Weighted |
| Summer | Winter | 25 | **0.001** | **0.001** | **0.001** | **0.001** |

| **Supplementary Table 9.**  Alpha diversity assessment of the sea urchin Diadema antillarum (black) across summer (N = 7) and winter (N = 18) samples (N = 25 total). Chao1, Faith PD, Shannon and Pielou and indices were compared with Kruskal–Wallis tests.  *P*-values are shown, with significant results in bold. | | | | | | | |
| --- | --- | --- | --- | --- | --- | --- | --- |
|  |  | **Experimental Group 1** | **Experimental Group 2** | **Chao1**  ***p*-value** | **Shannon**  ***p*-value** | **Faith PD**  ***p*-value** | **Pielou’s**  ***p*-value** |
| **Black species per season**  **(N=25)** | Summer (N=7) | Winter (N=18) | 25 | **0.000** | **0.011** | **0.001** | 0.102 |
