# Supplementary figures and images for: Seasonality shapes gut microbiota composition in two sympatric sea urchins
Source: PeerJ. 2026 Mar 5;14:e20918. doi: 10.7717/peerj.20918 (PMC12967421; doi:10.7717/peerj.20918)

# A) Unifrac Unweighted

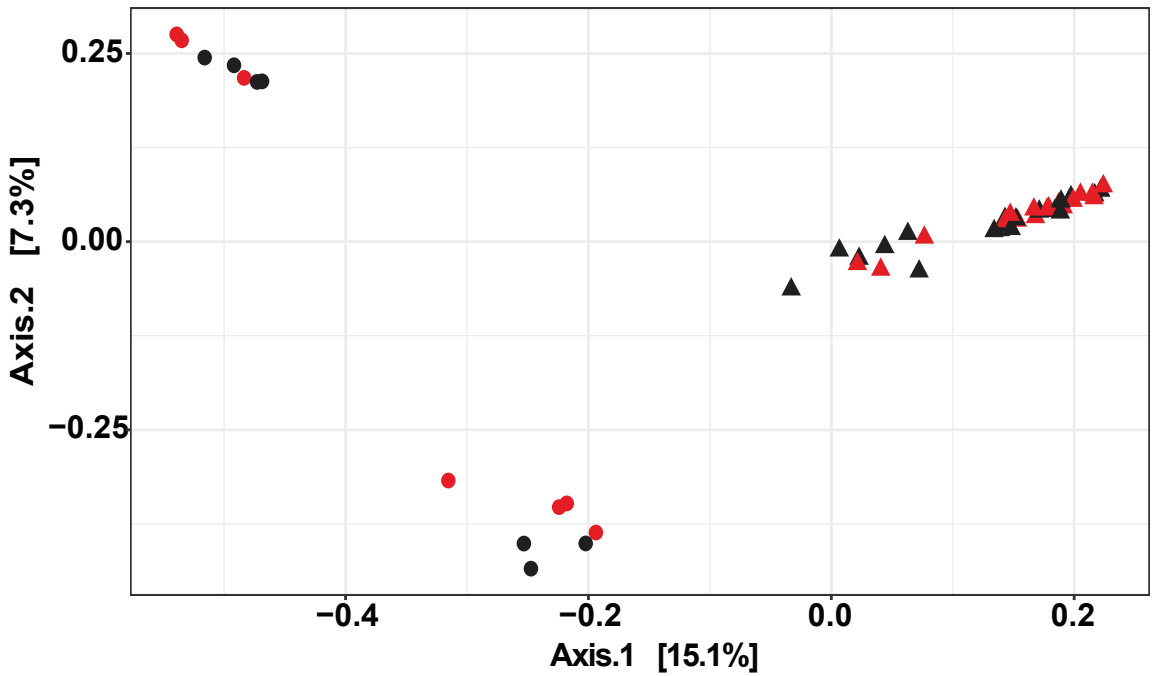

# B) Unifrac Weighted

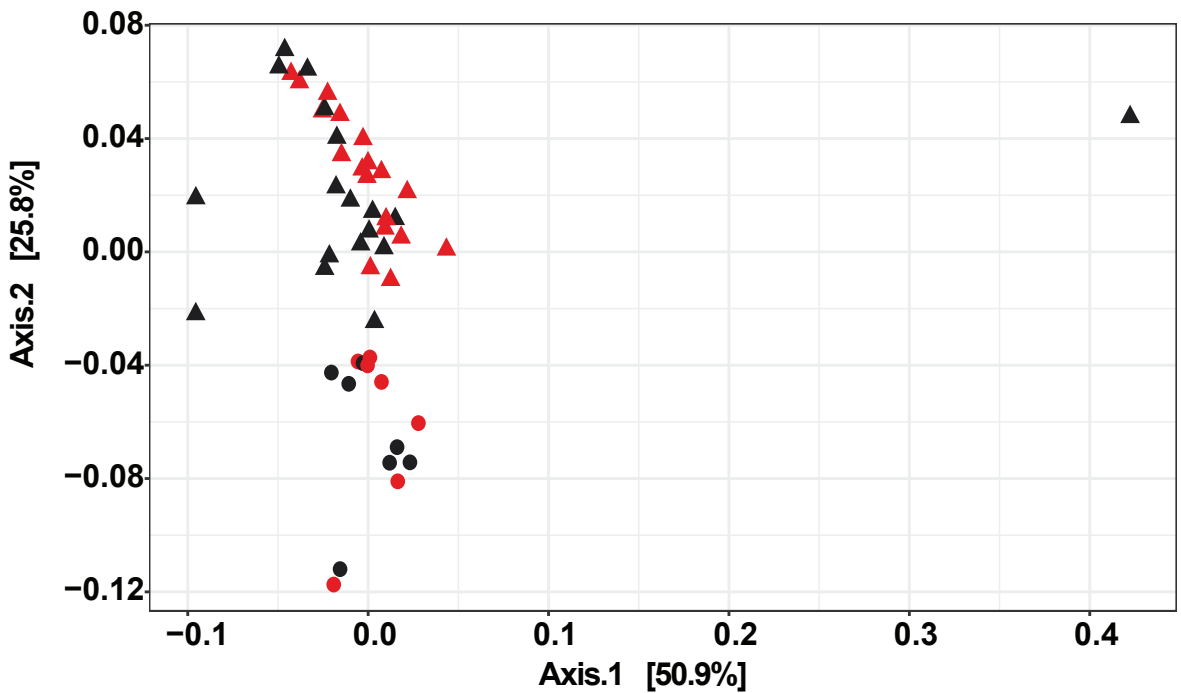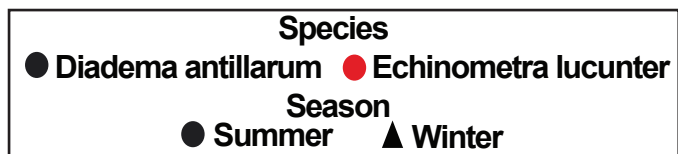

Supplement: Supplemental Information 2 [file peerj-14-20918-s002.pdf]

## A) Unifrac Unweighted

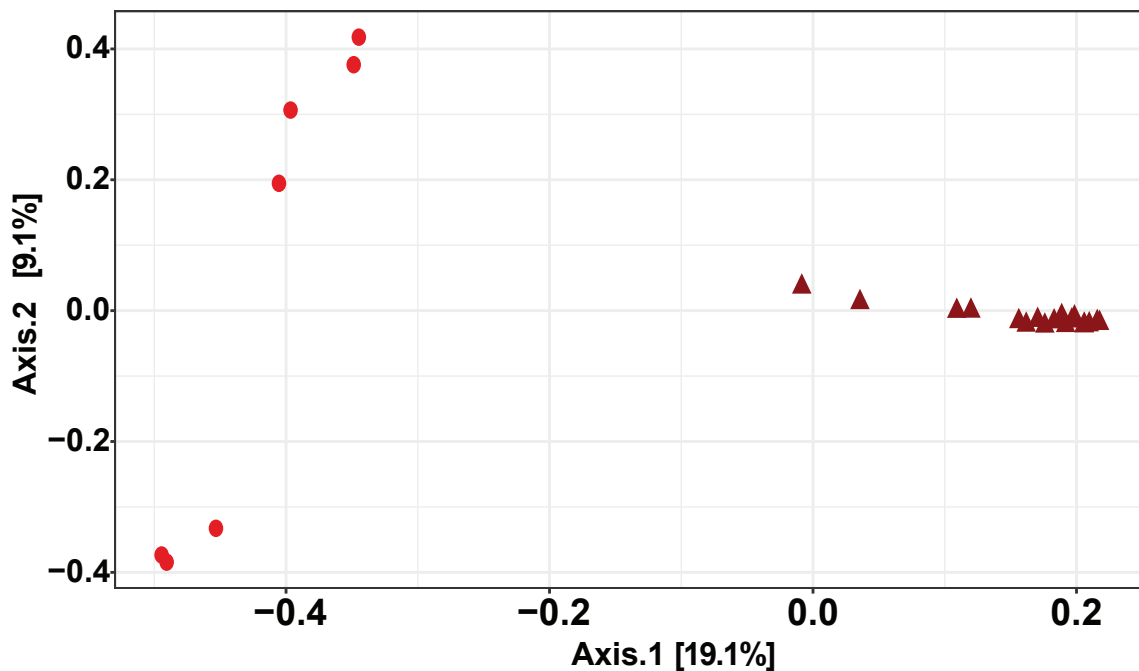

## B) Unifrac Weighted

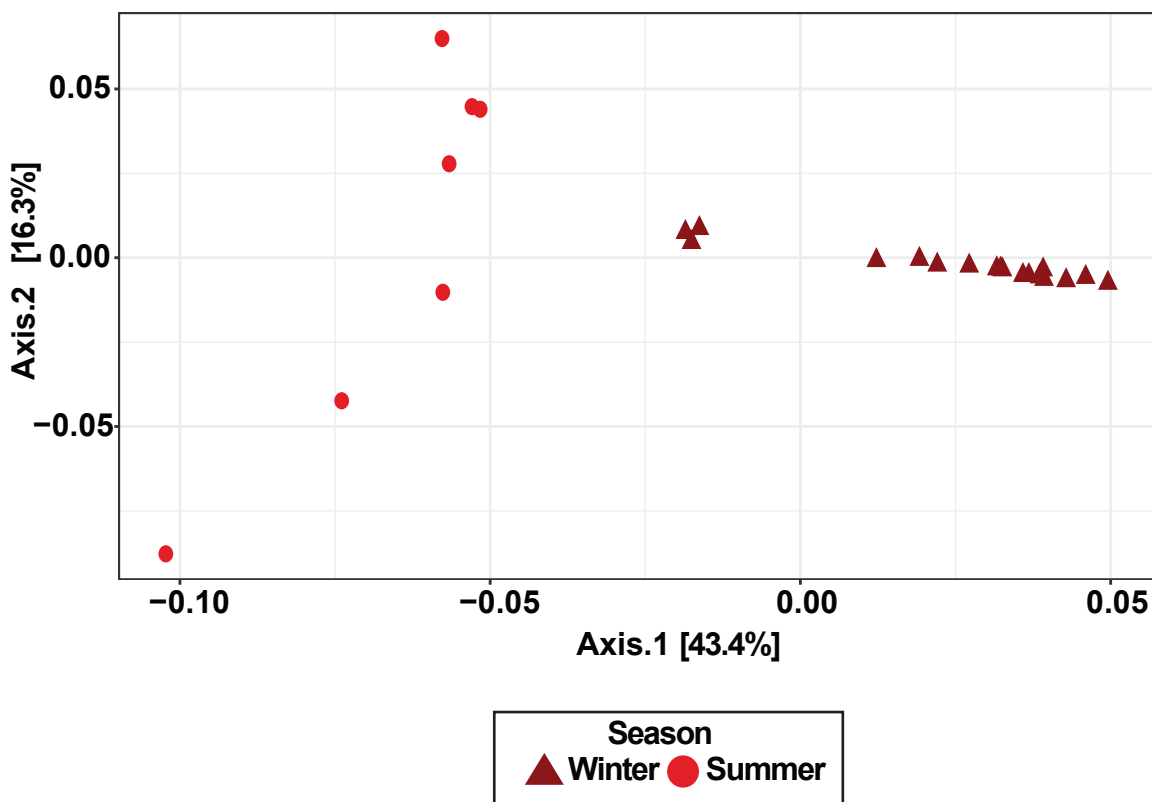

Supplement: Supplemental Information 3 [file peerj-14-20918-s003.pdf]

A)

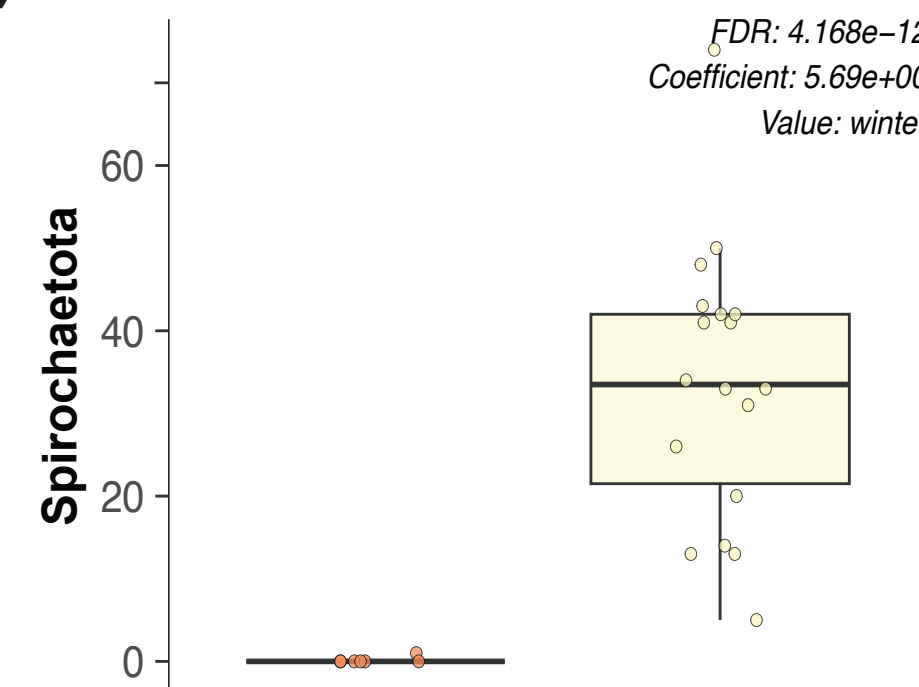

B)

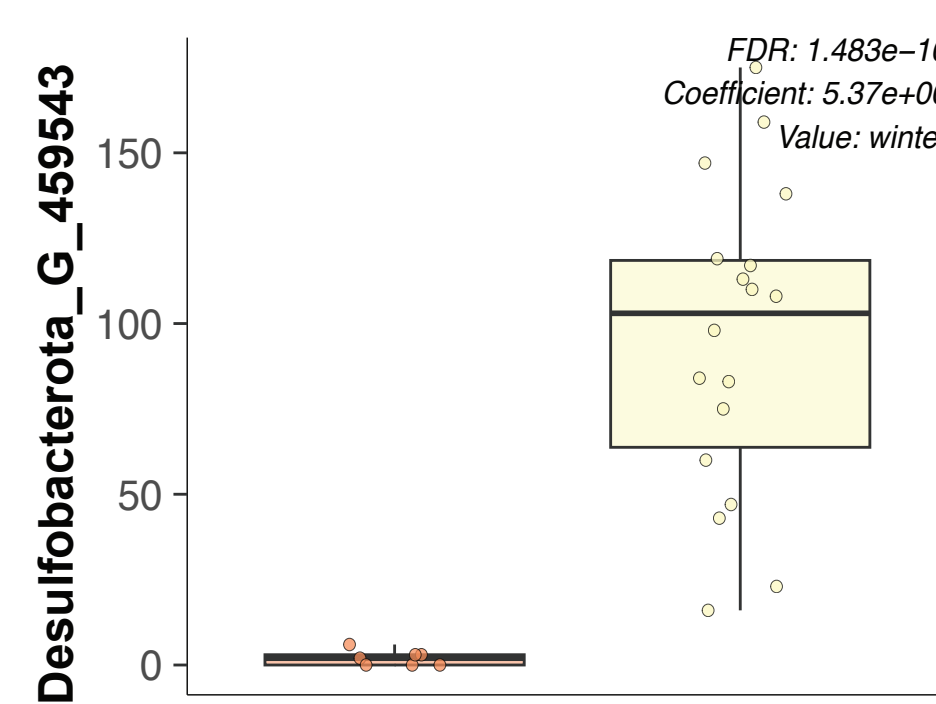

C)

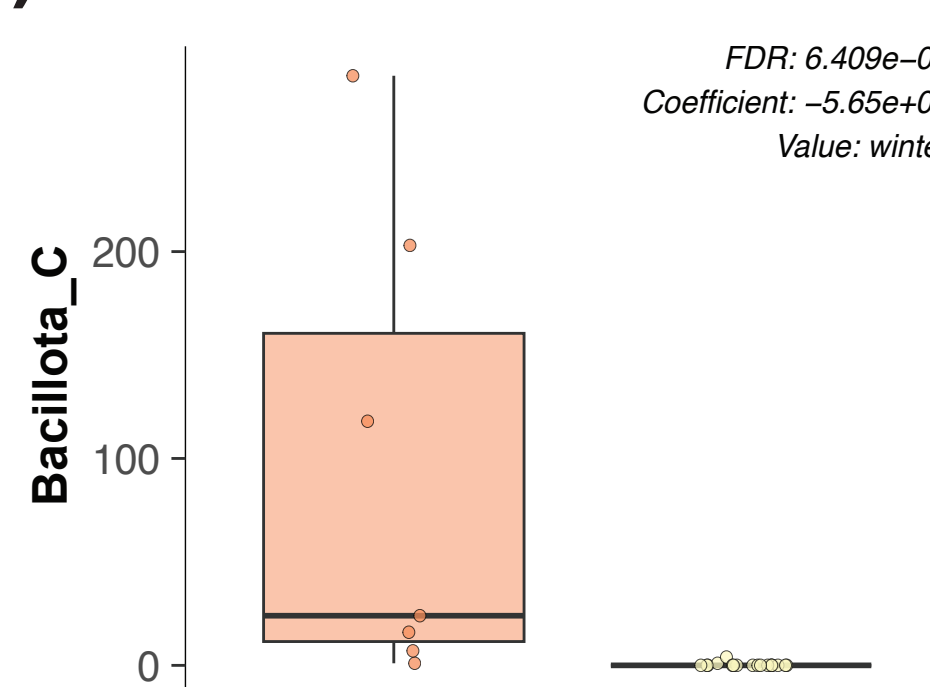

D)

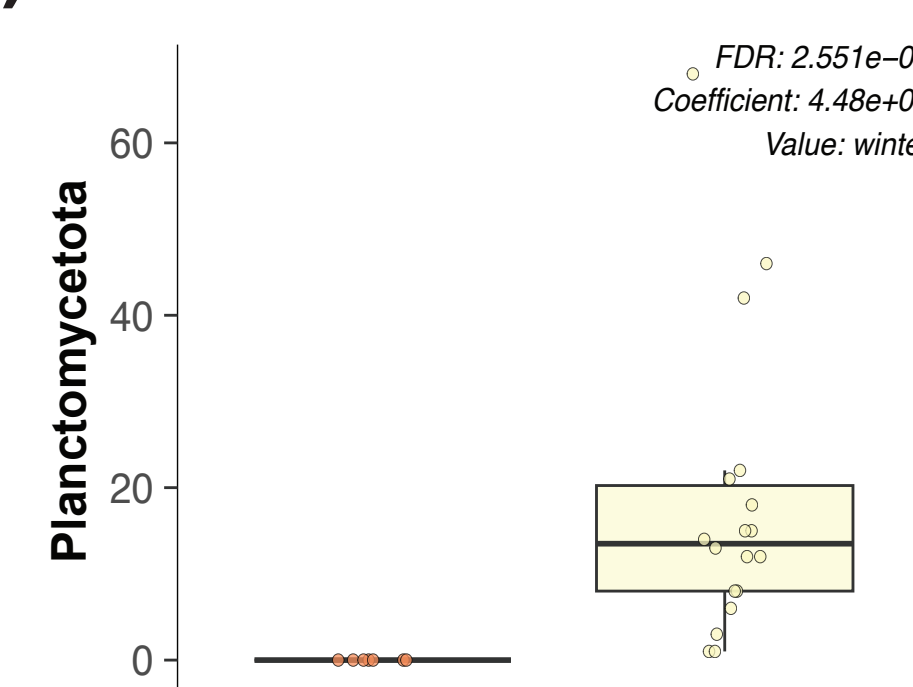

E)

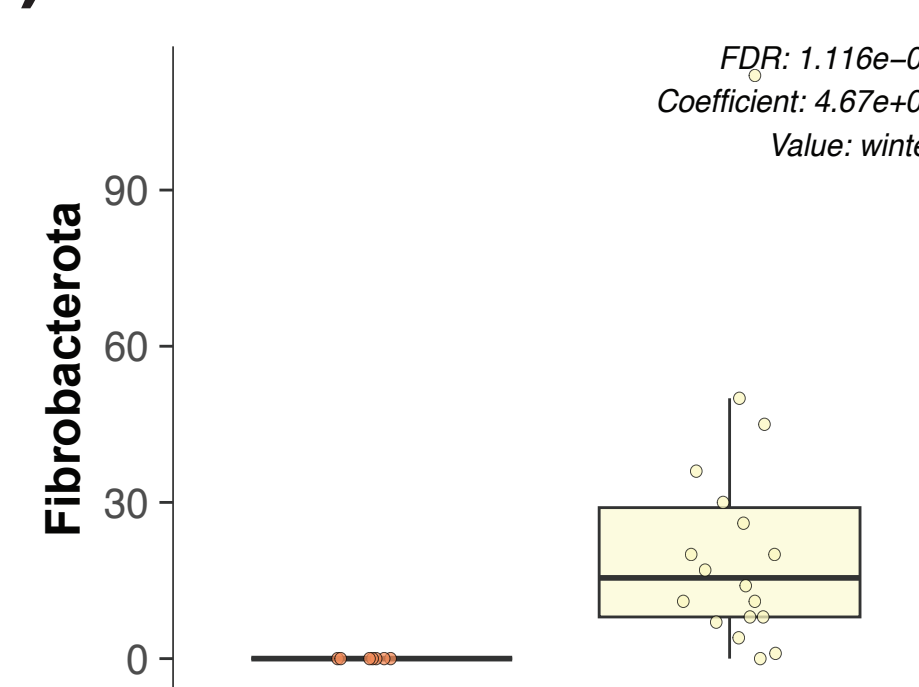

F)

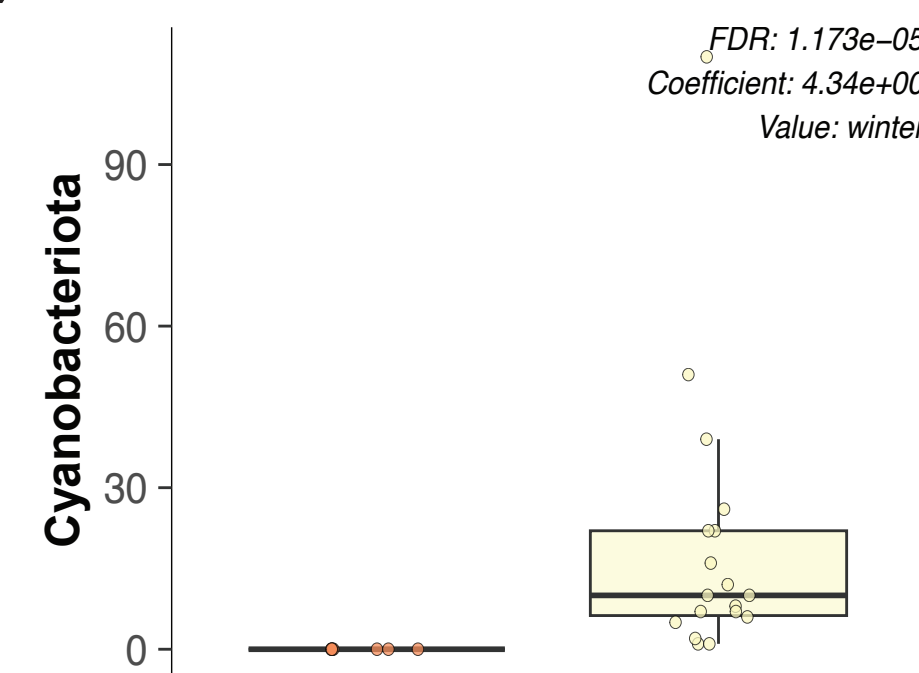

G)

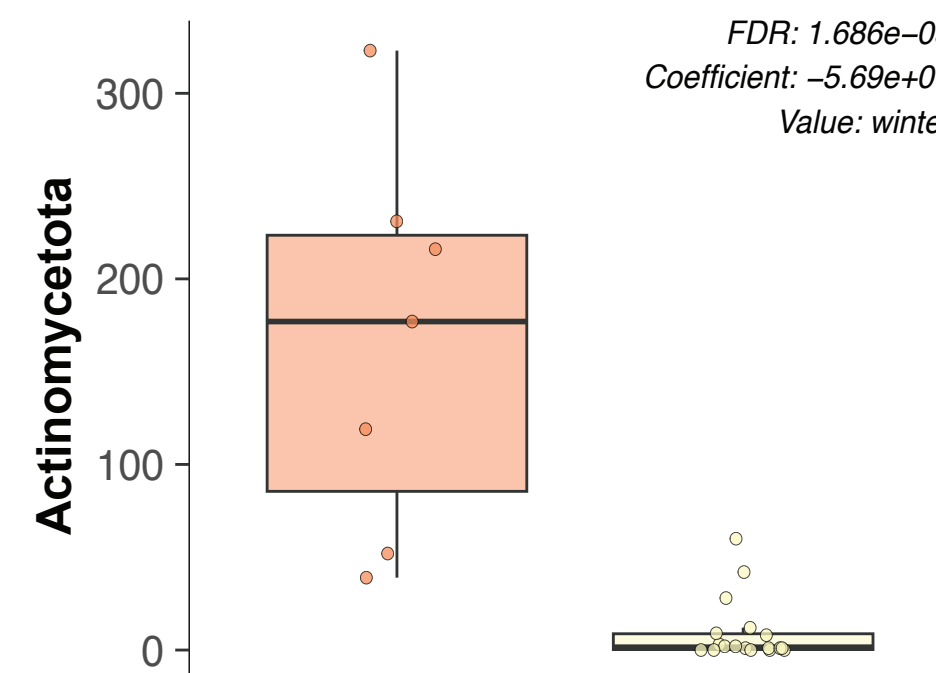

H)

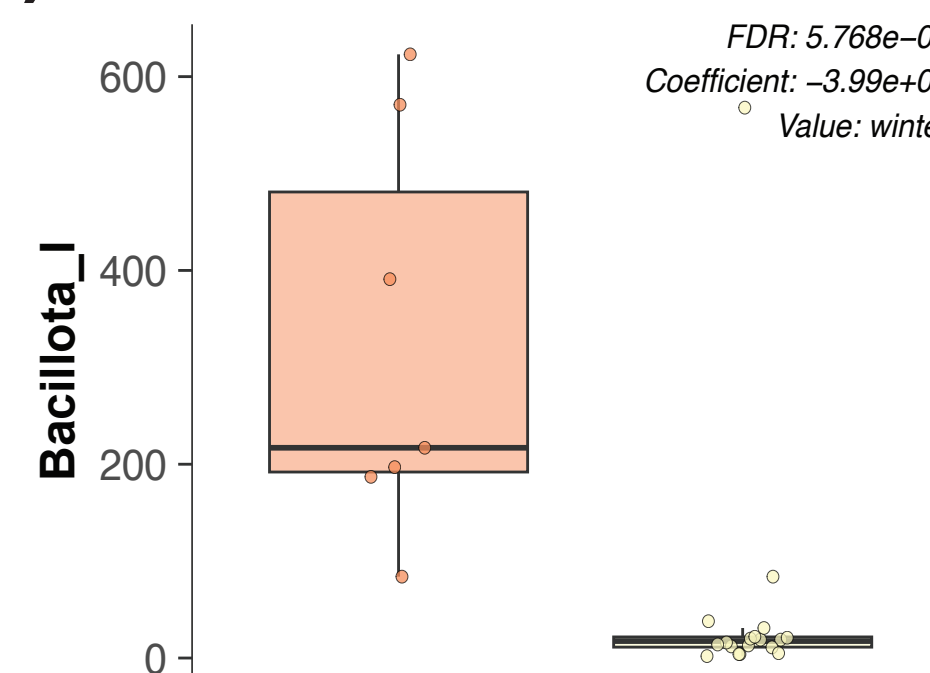

I)

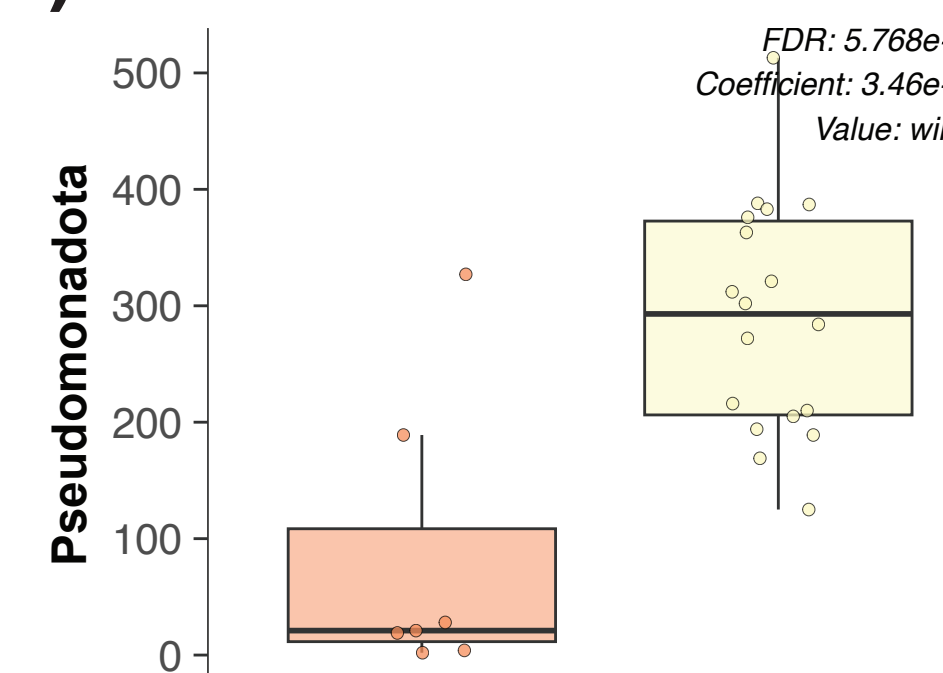

J)

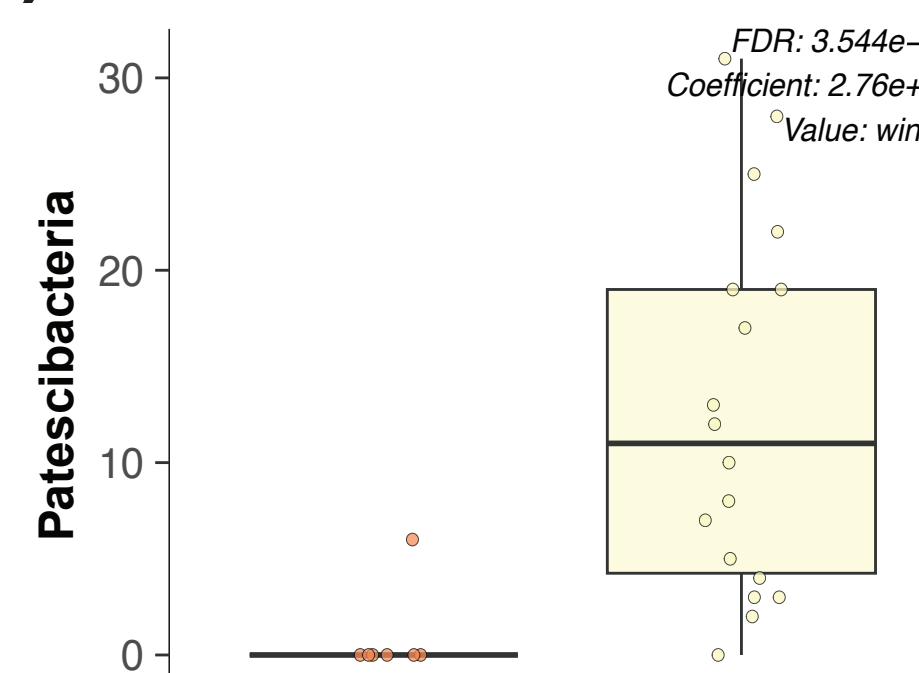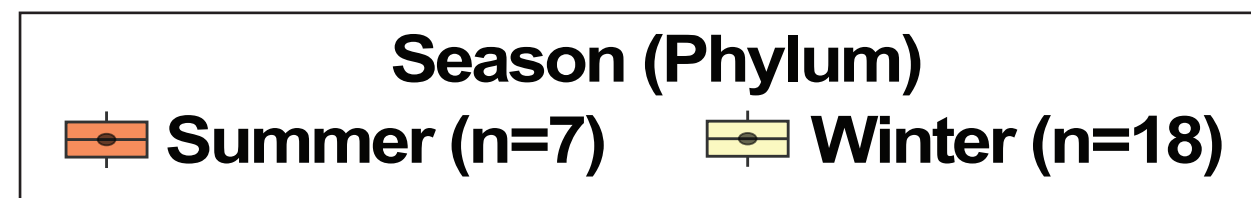

Supplement: Supplemental Information 4 [file peerj-14-20918-s004.pdf]

A)

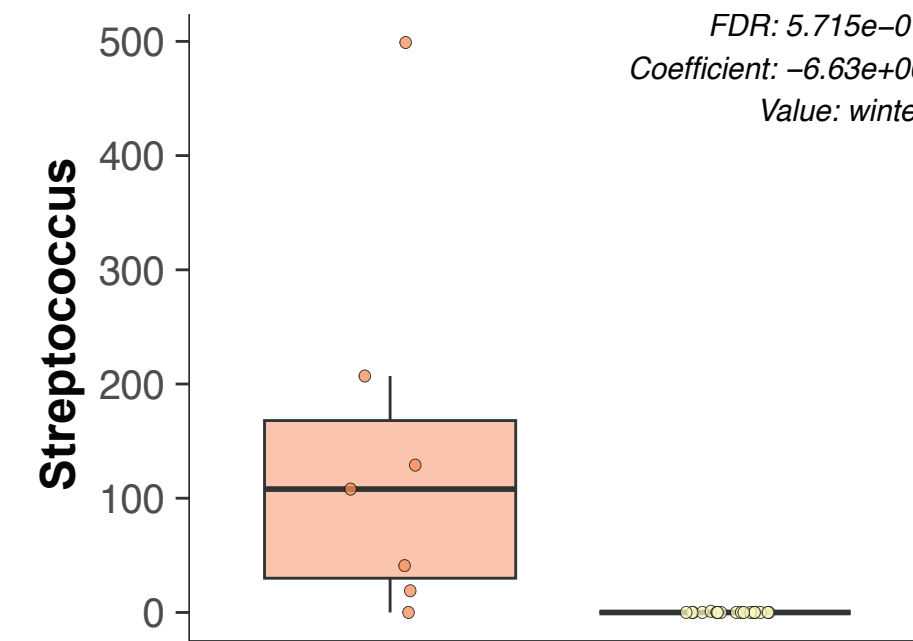

B)

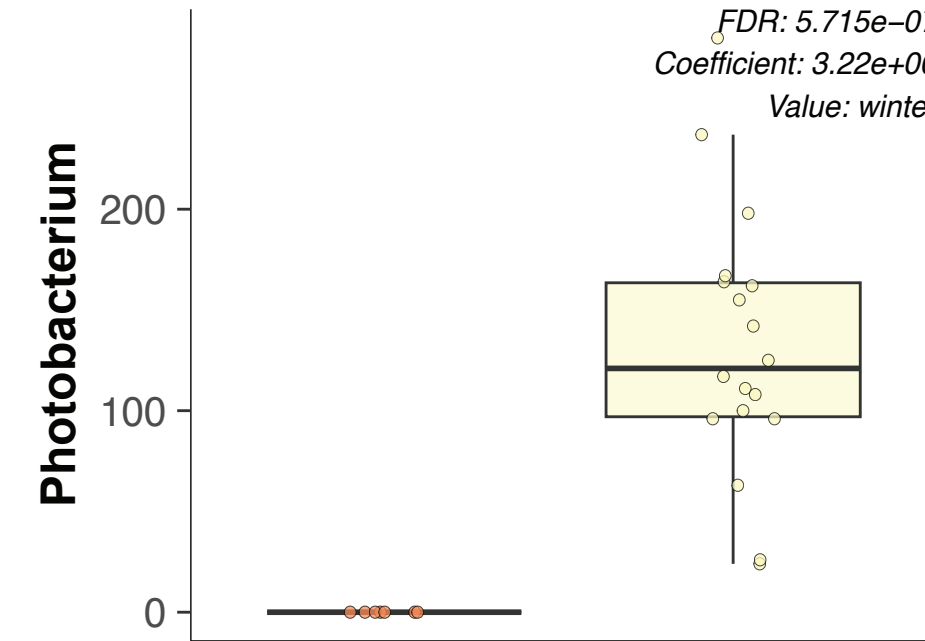

C)

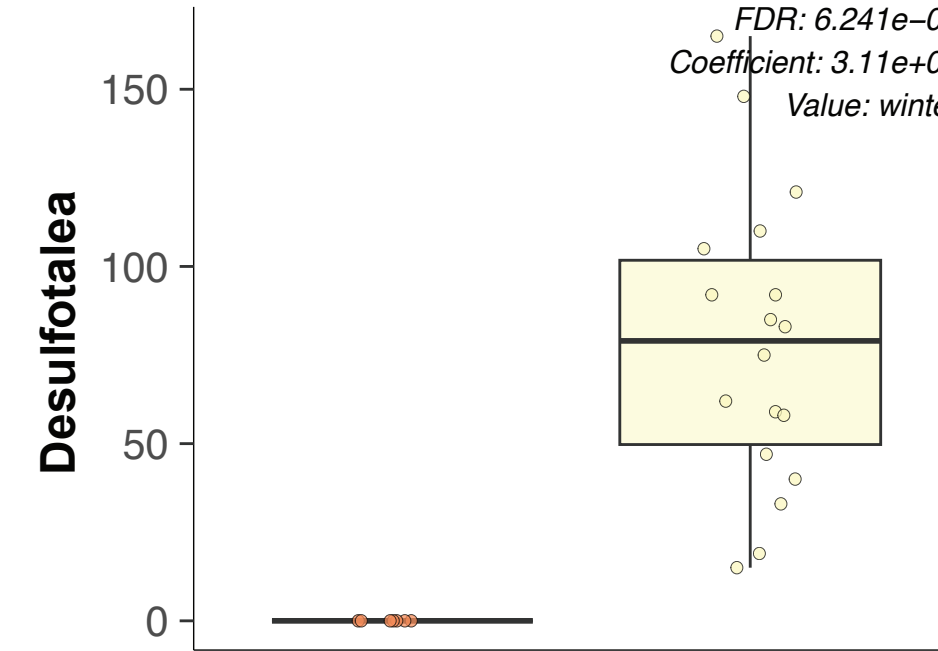

D)

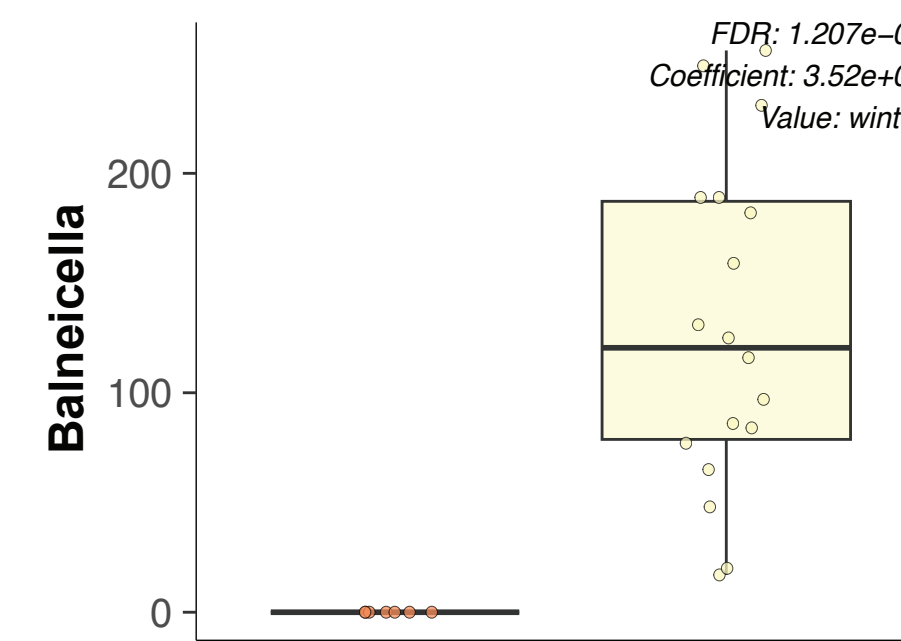

E)

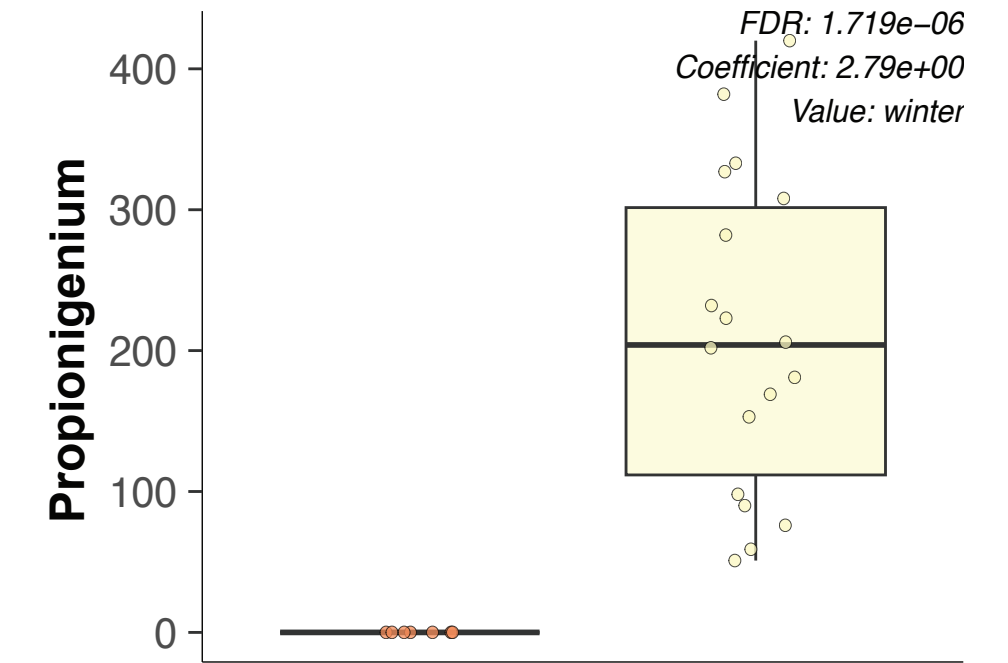

F)

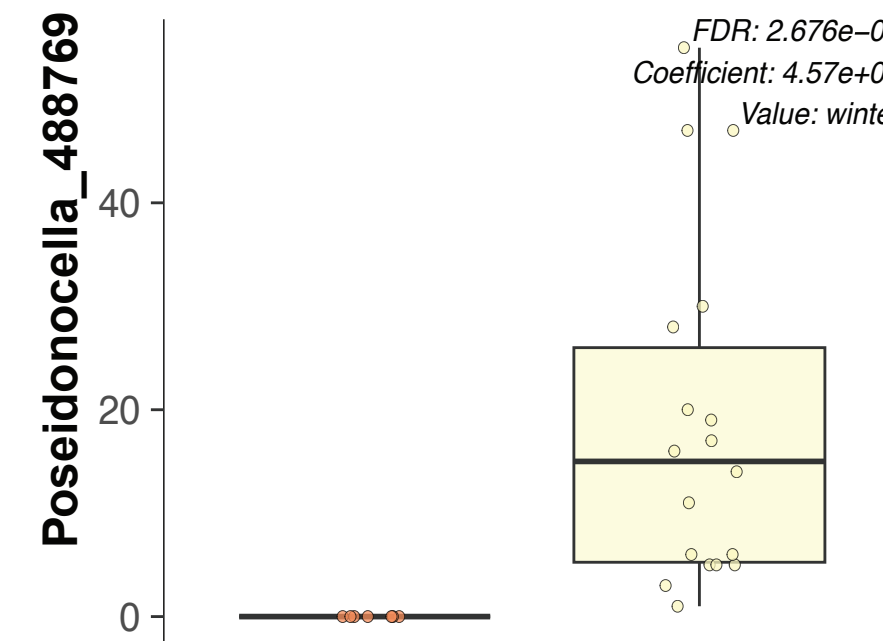

G)

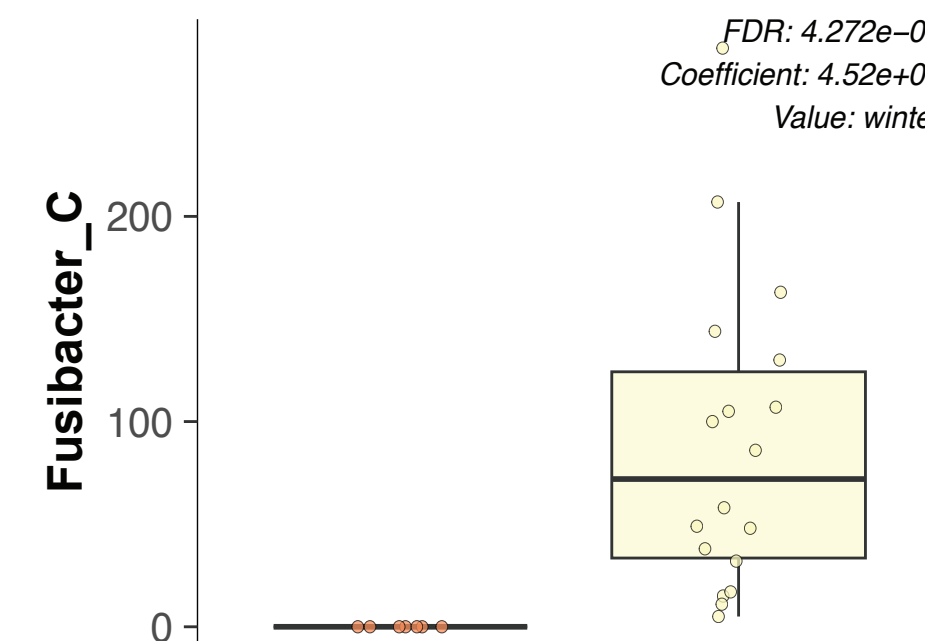

H)

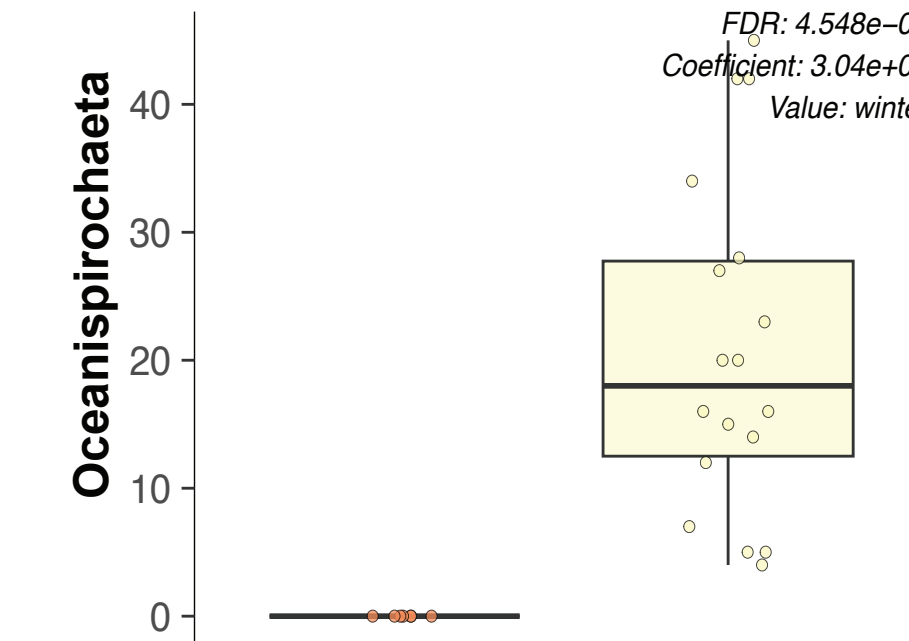

I)

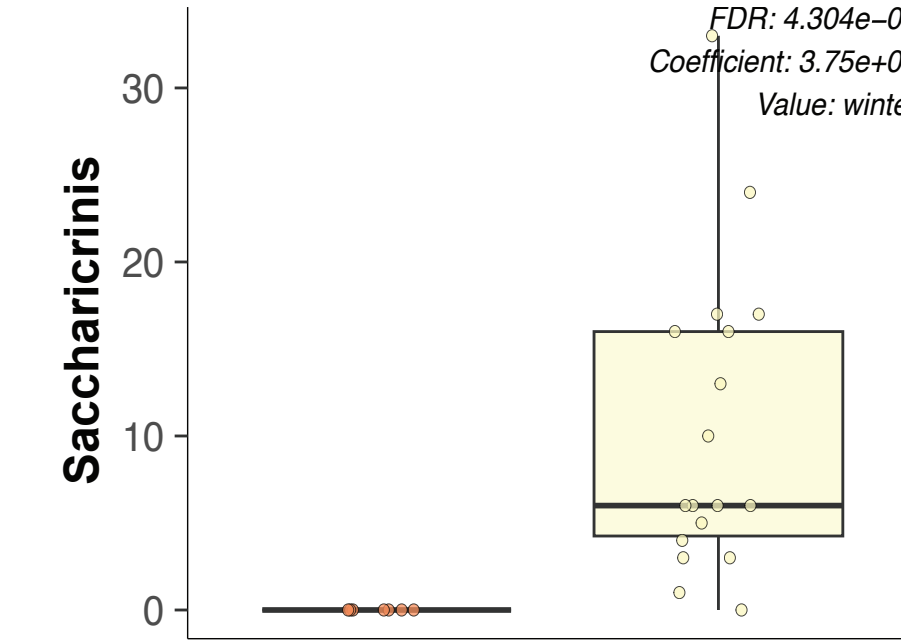

J)

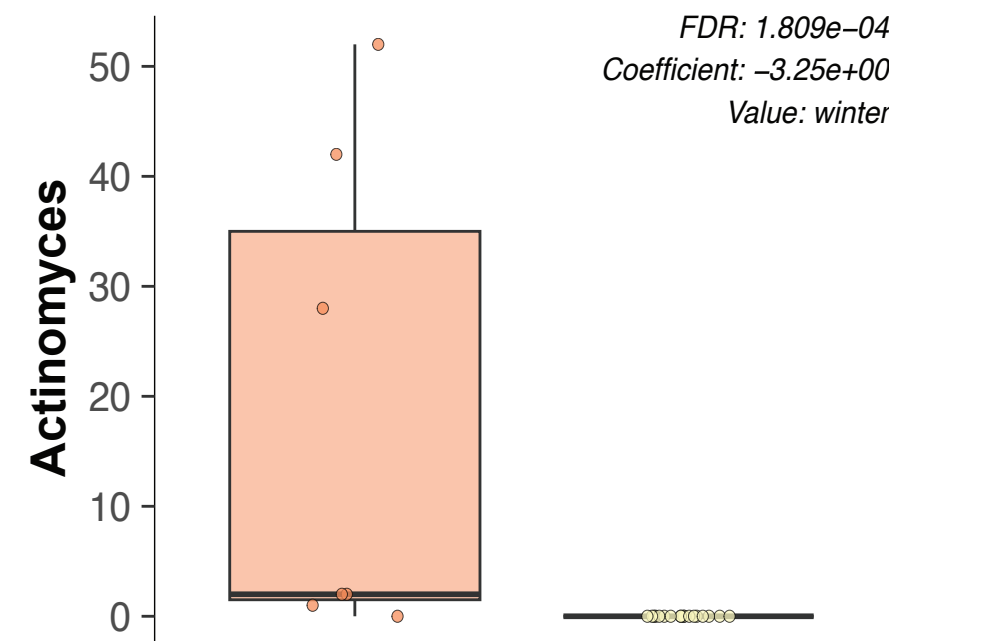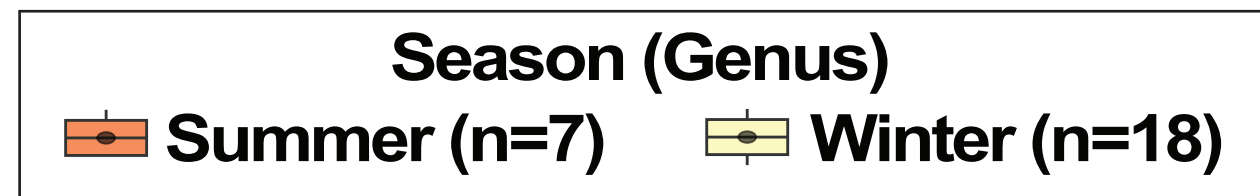

Supplement: Supplemental Information 5 [file peerj-14-20918-s005.pdf]

# A) Unifrac Unweighted

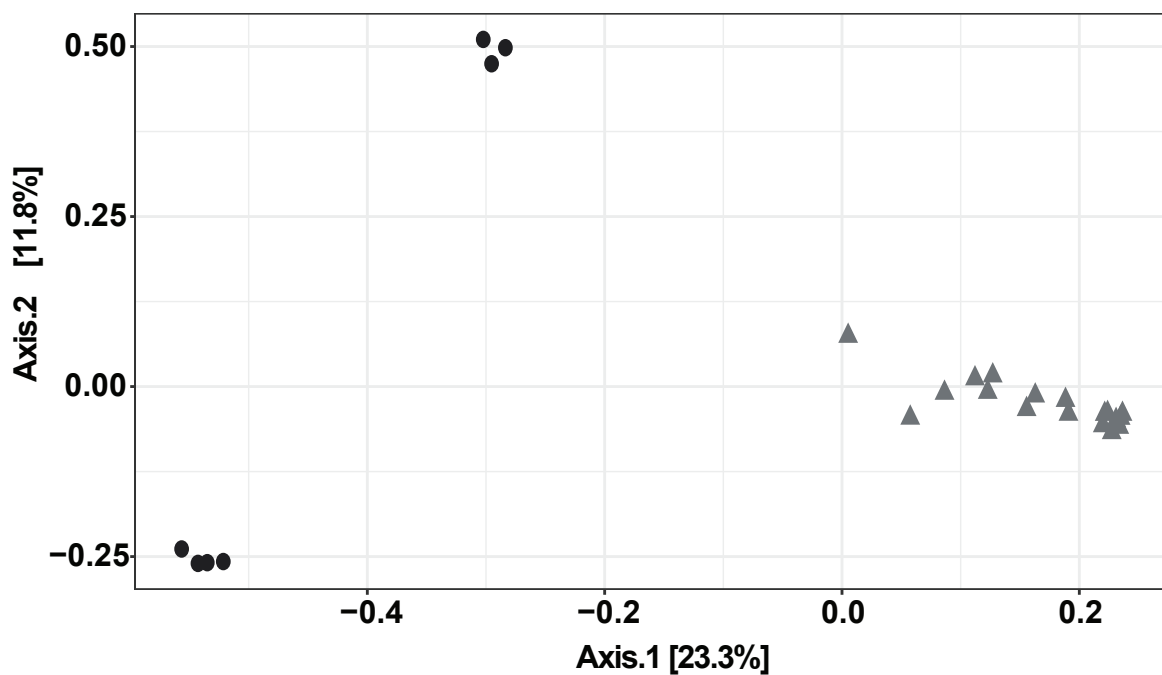

# B) Unifrac Weighted

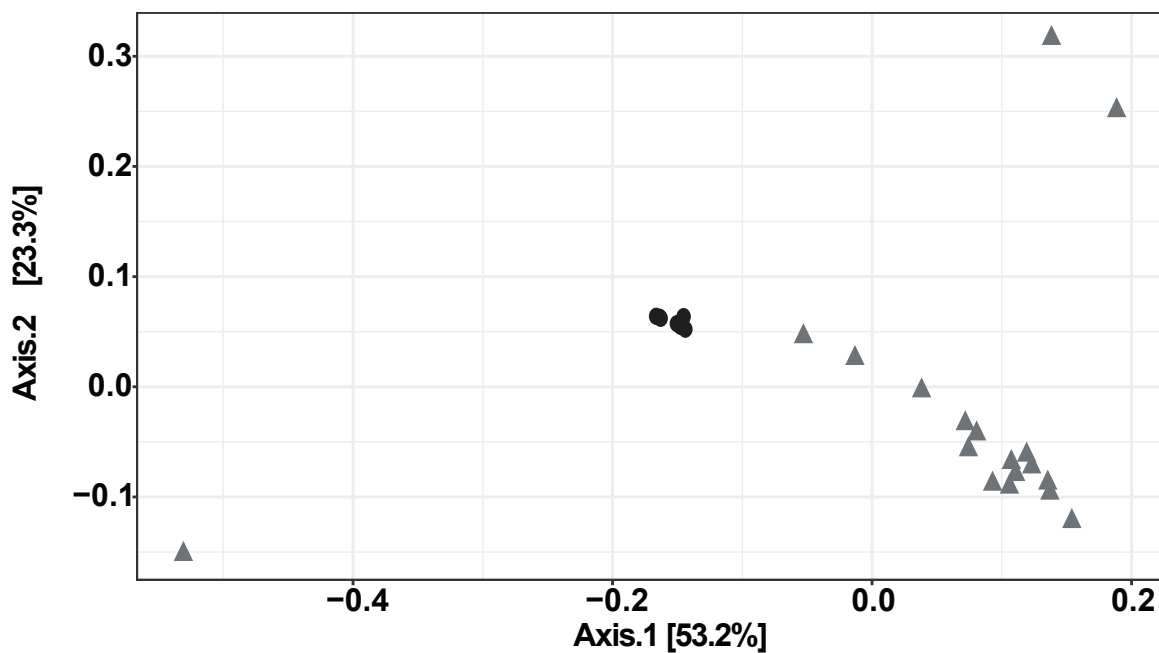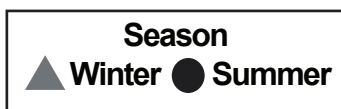

Supplement: Supplemental Information 6 [file peerj-14-20918-s006.pdf]

A)

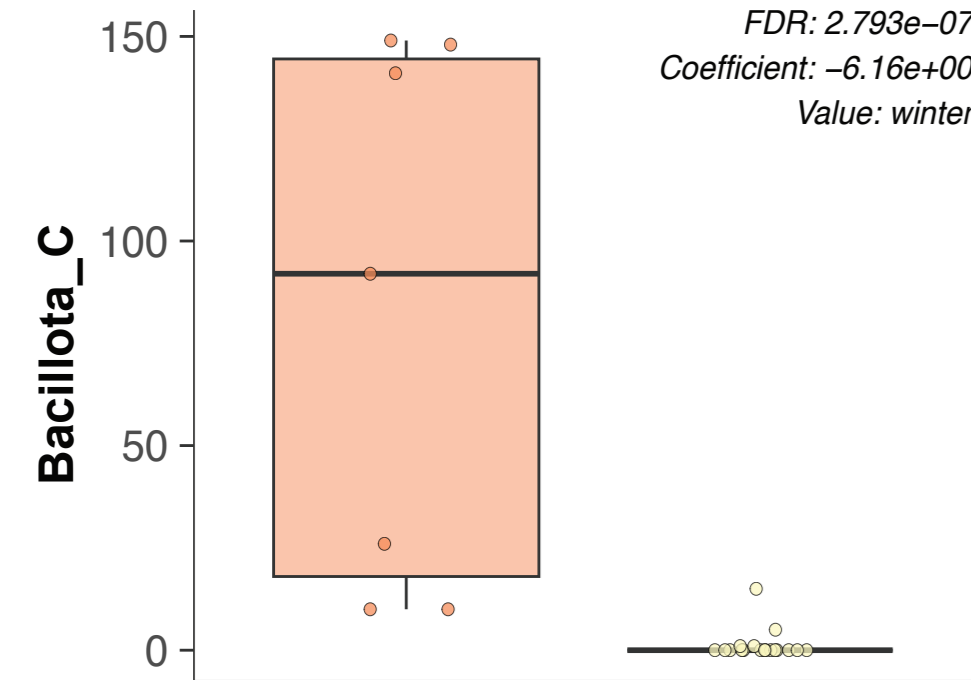

B)

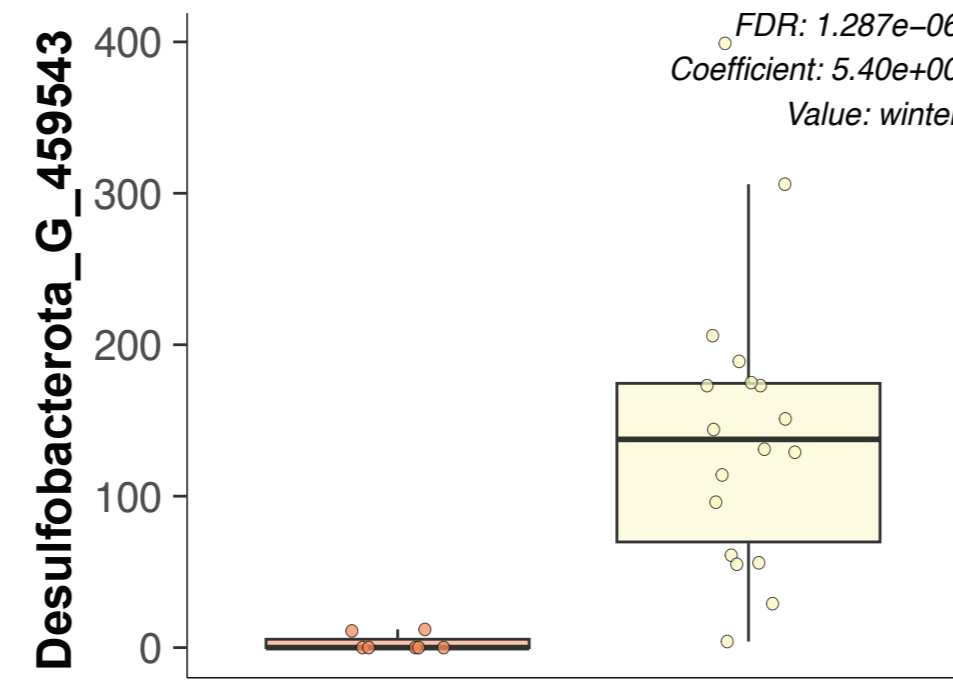

C)

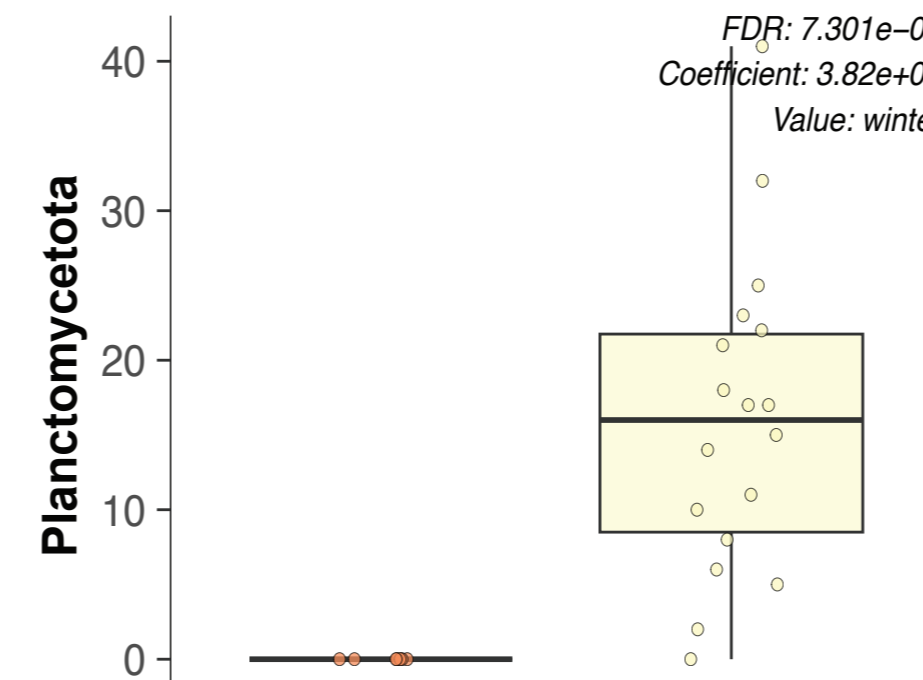

D)

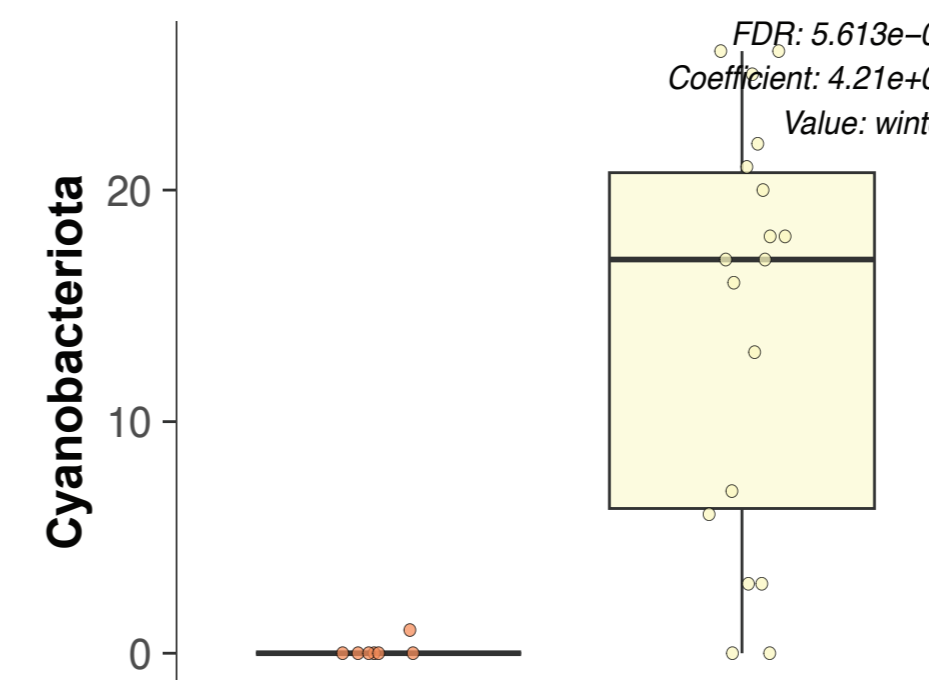

E)

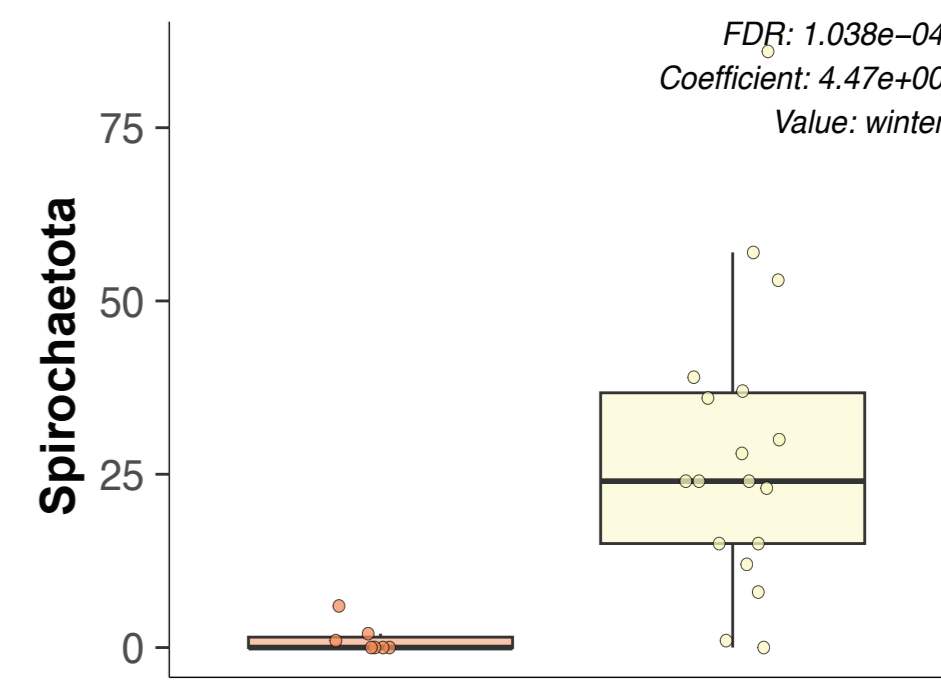

F)

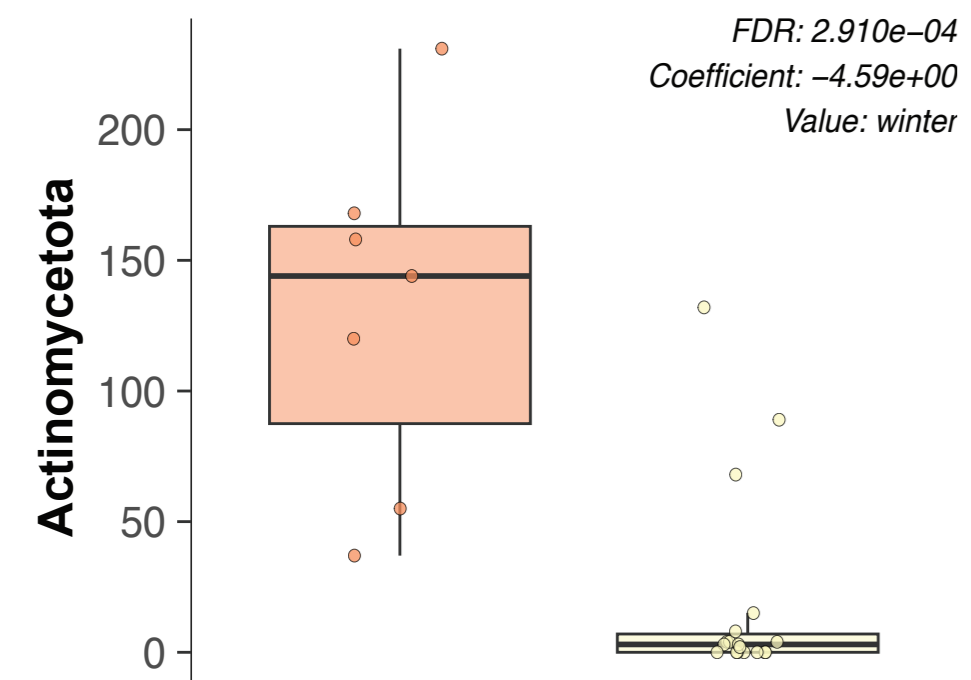

G)

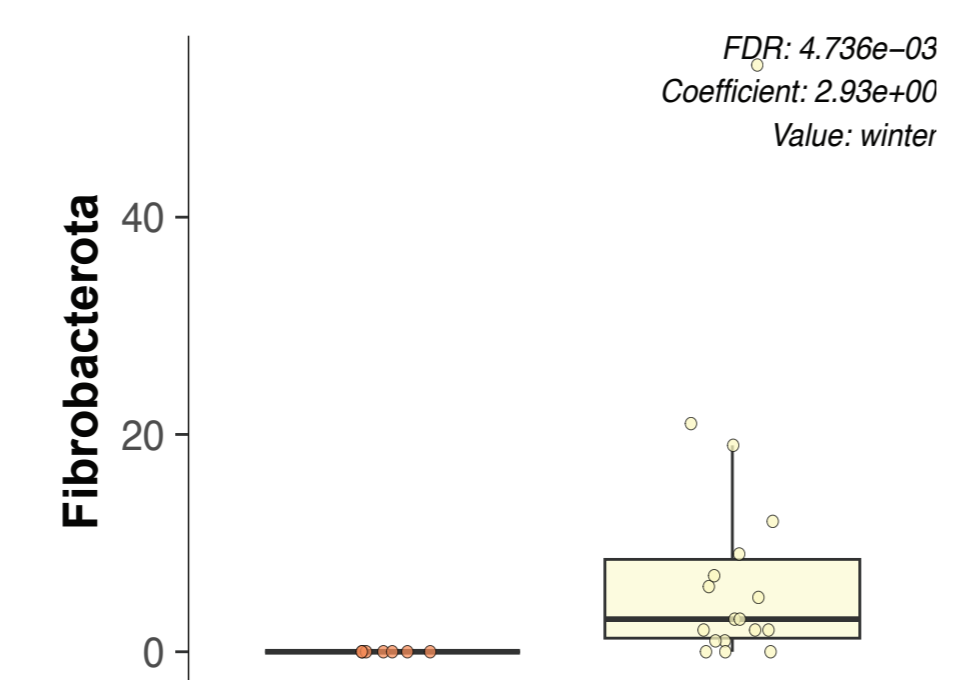

H)

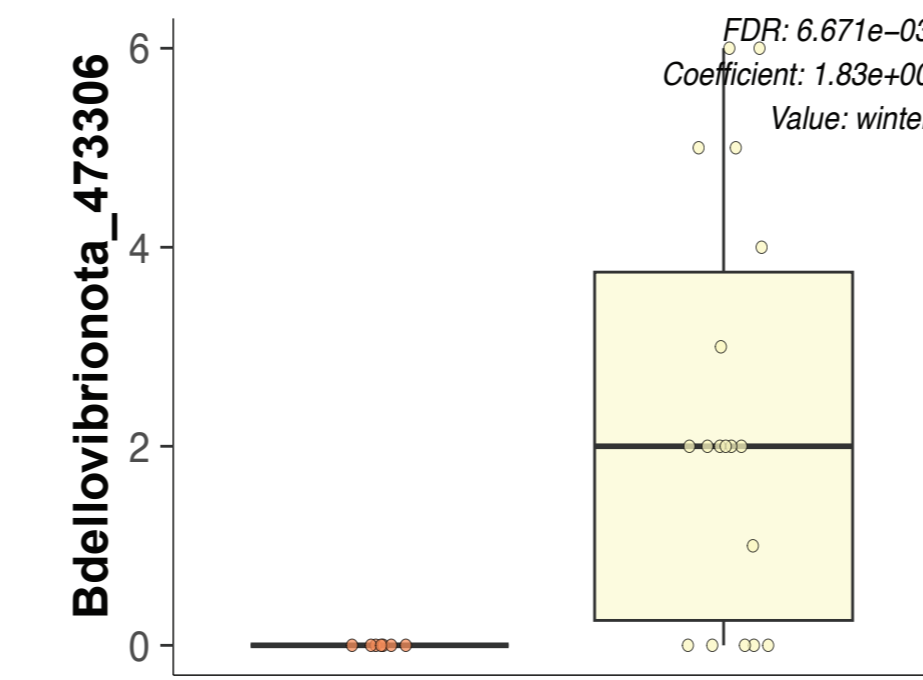

I)

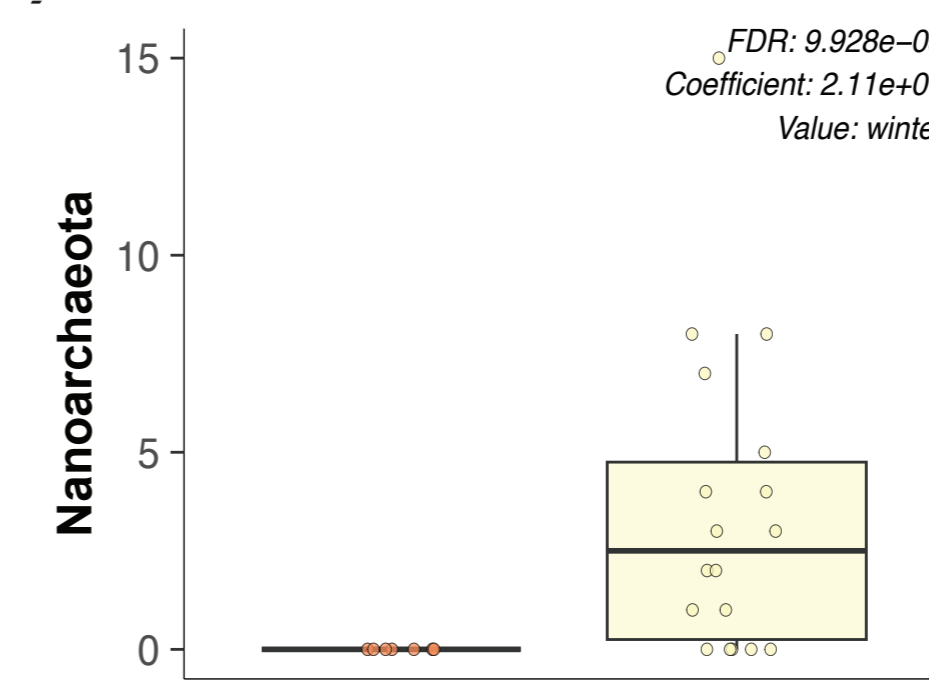

J)

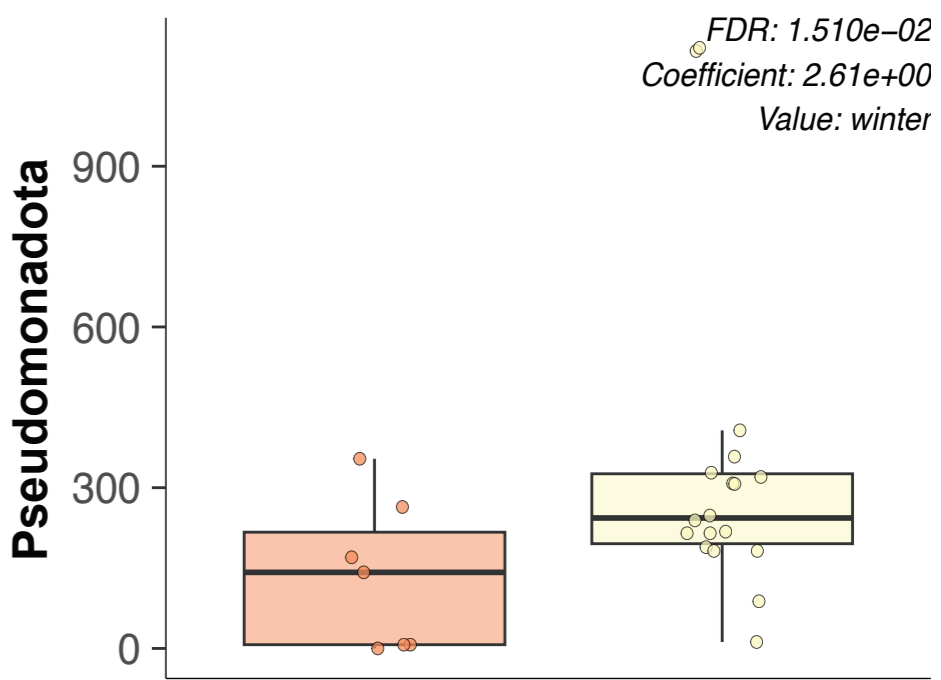

Season (Phylum)

Summer (n=7)    Winter (n=18)

Supplement: Supplemental Information 7 [file peerj-14-20918-s007.pdf]

A)

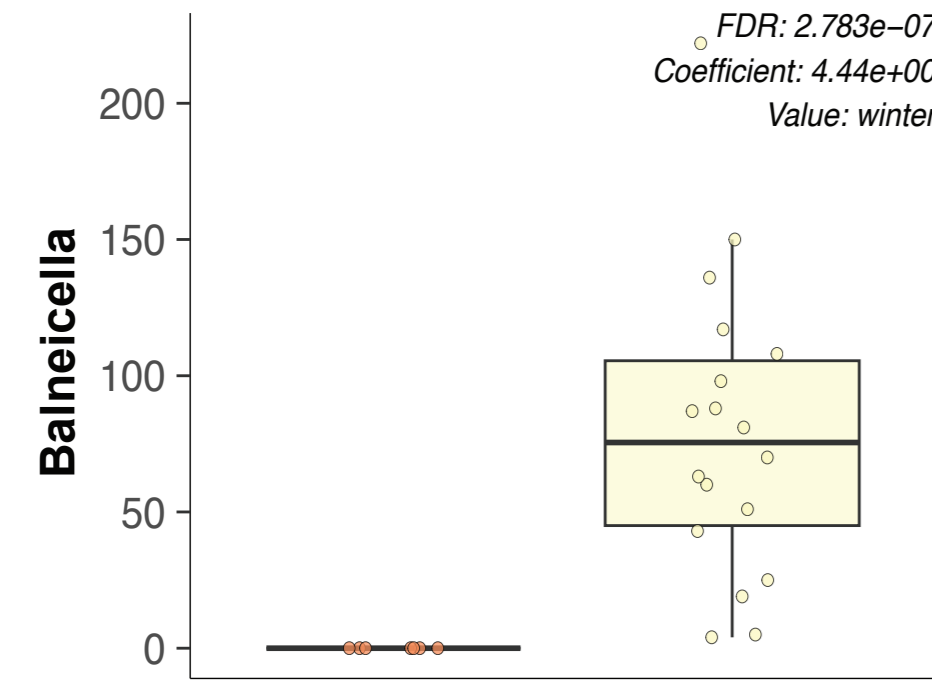

B)

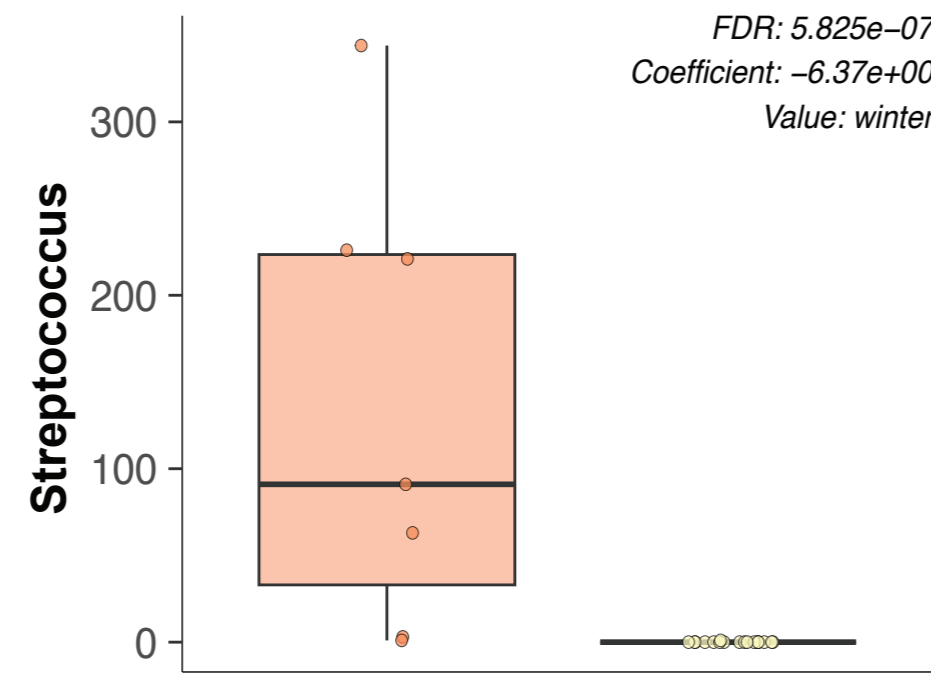

C)

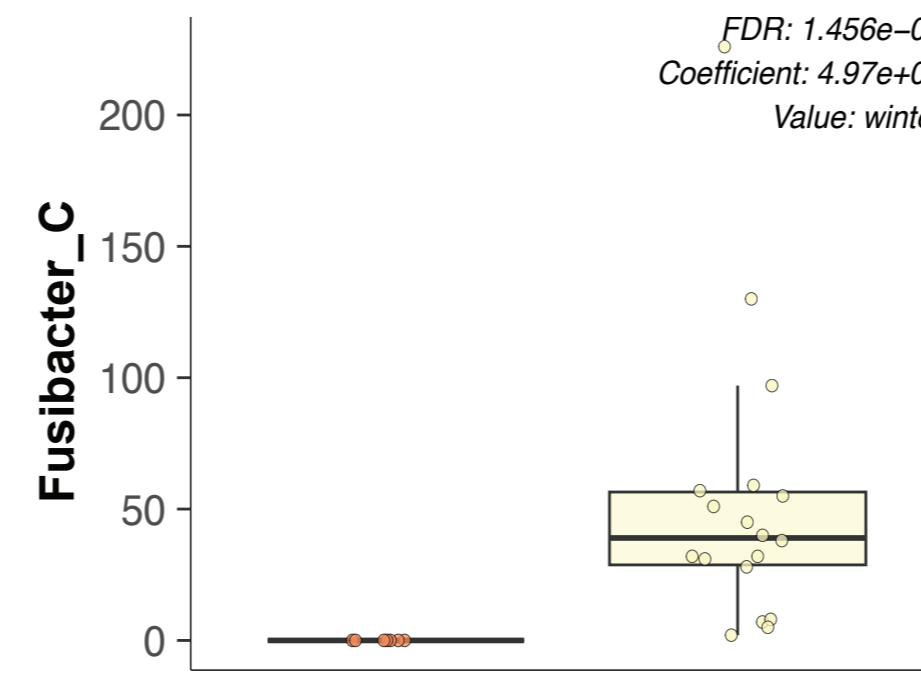

D)

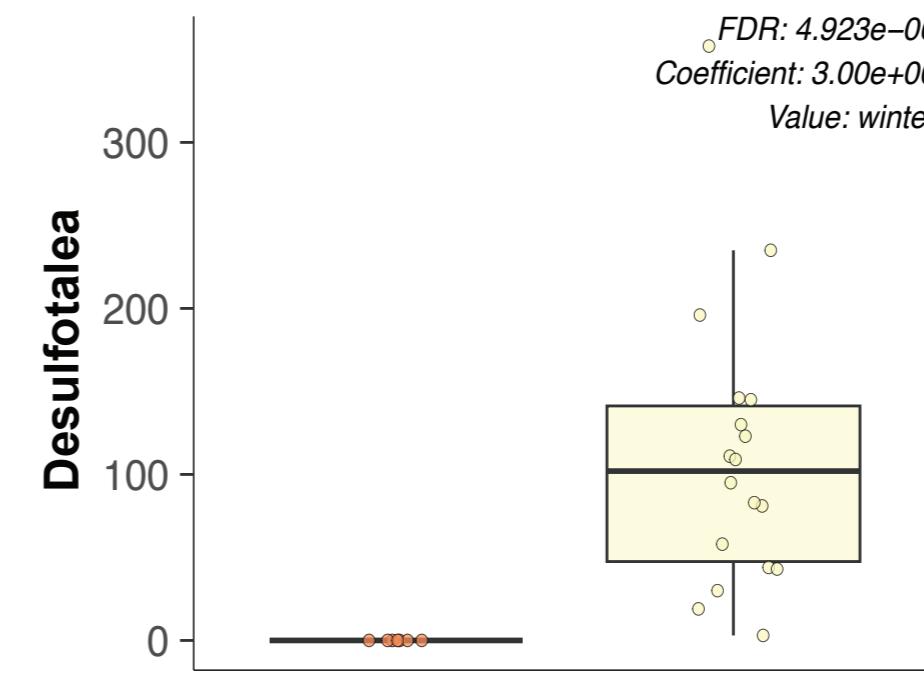

E)

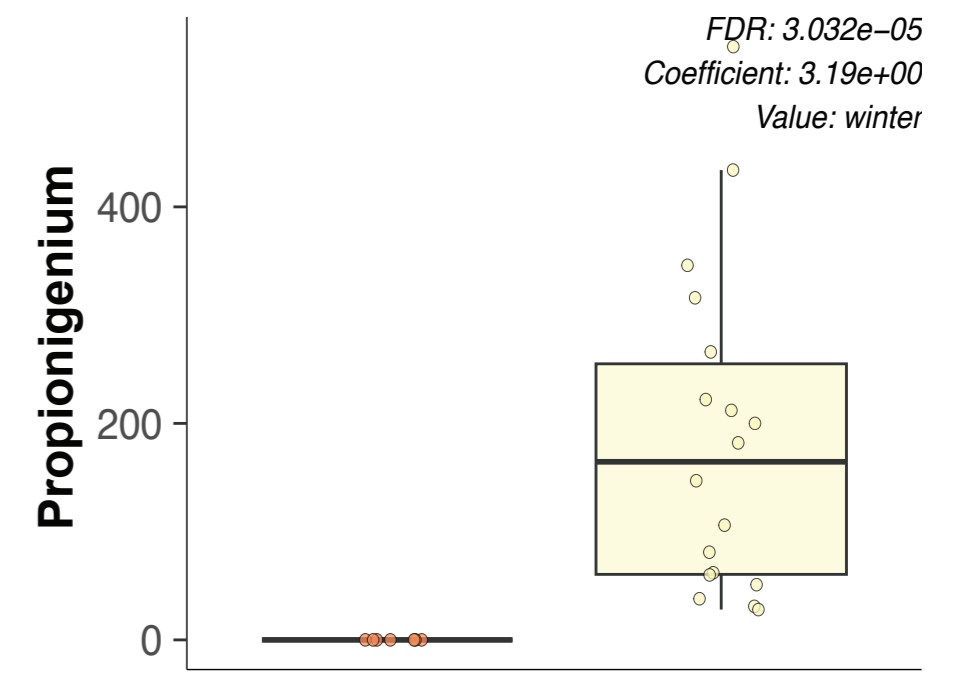

F)

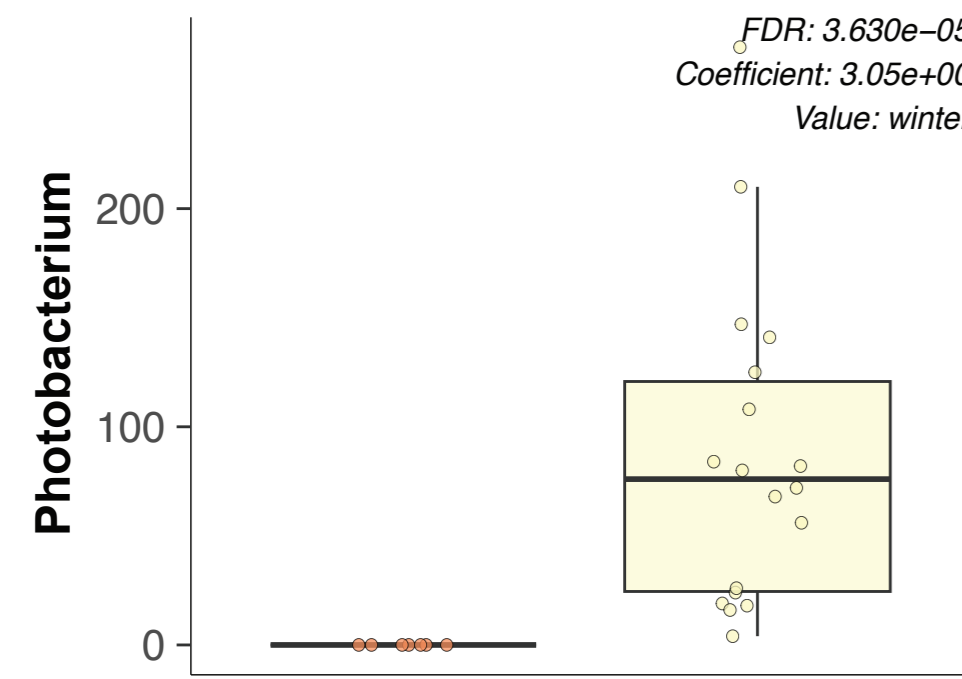

G)

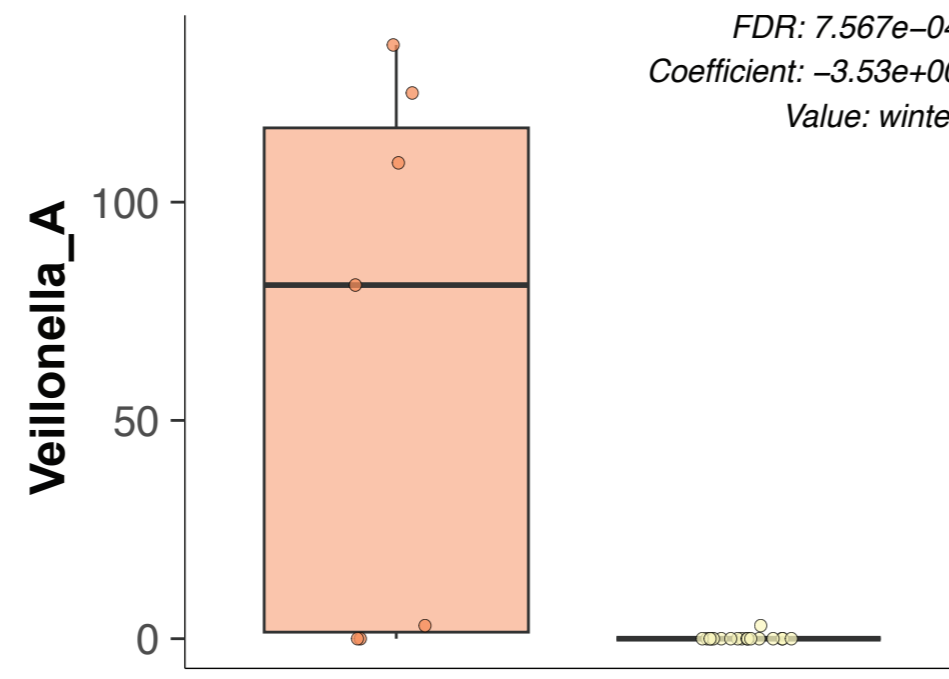

H)

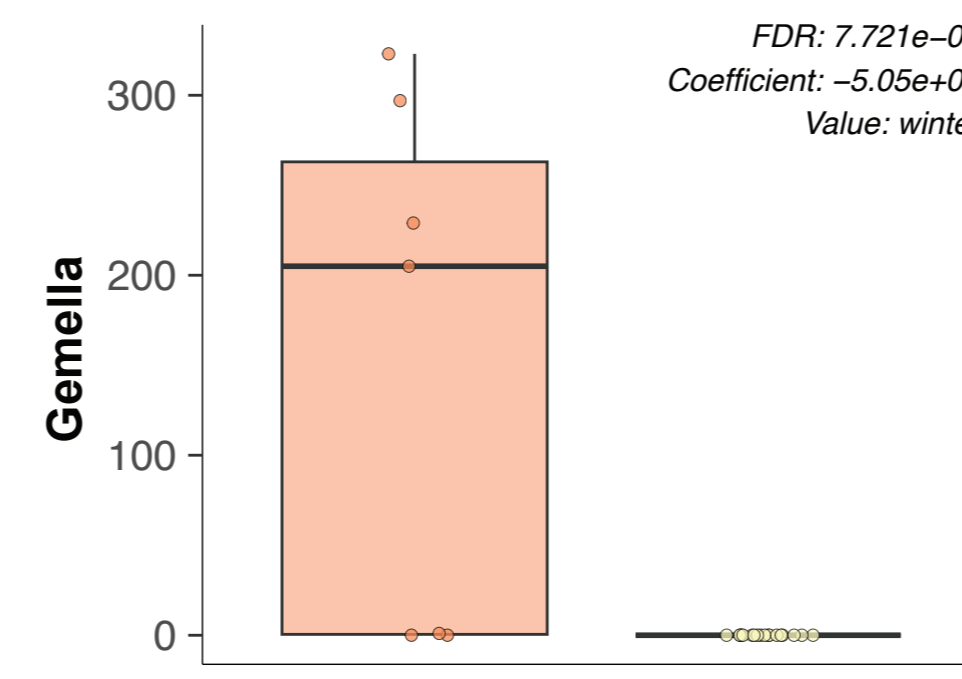

I)

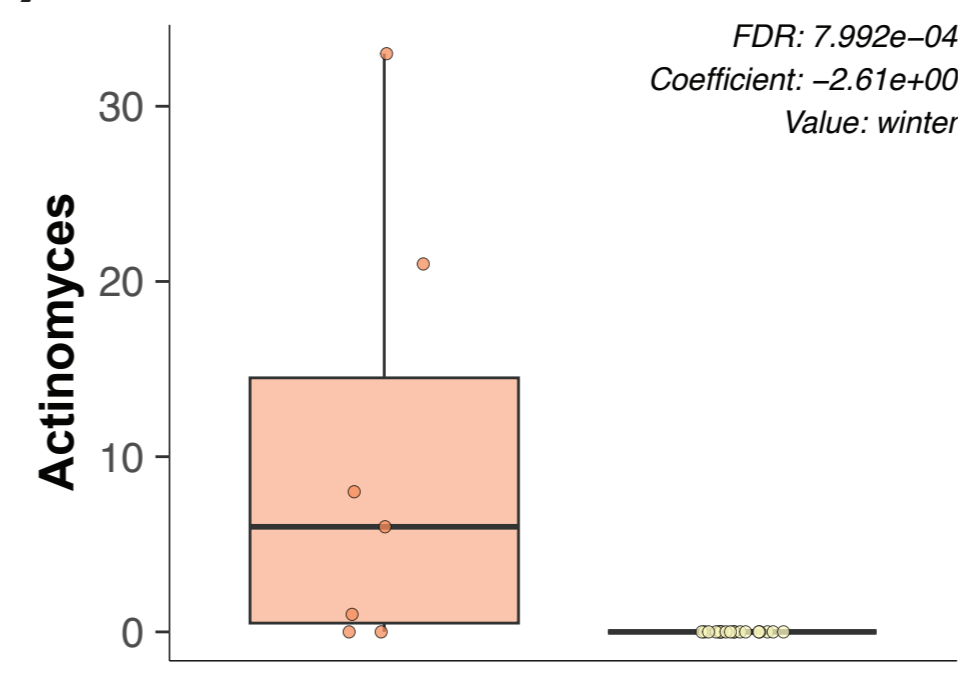

J)

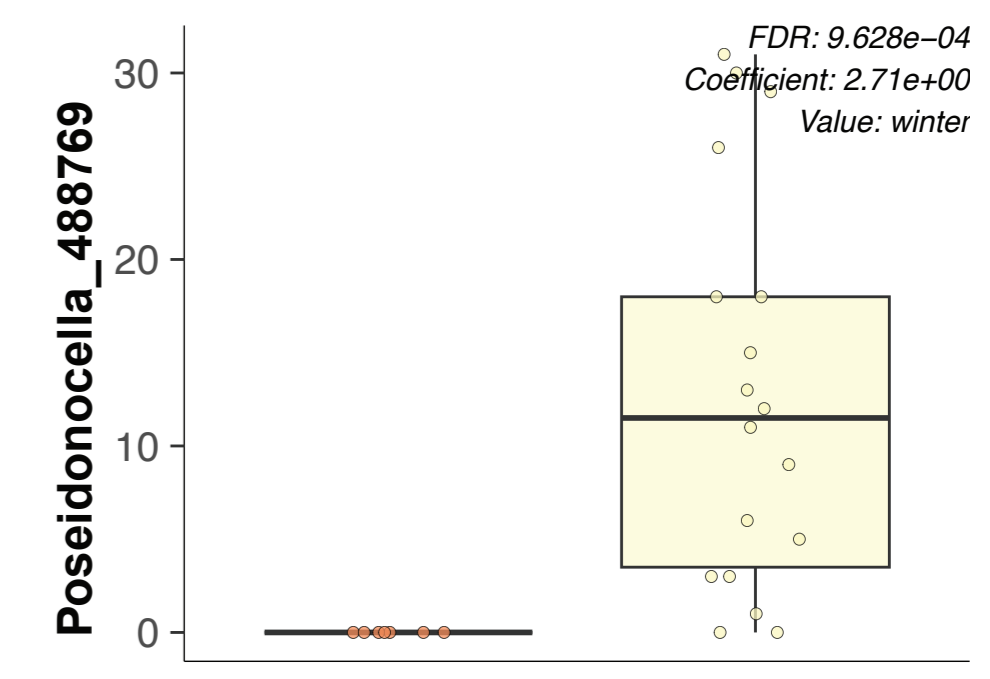

Season (Genus)

Summer (n=7) Winter (n=18)

Supplement: Supplemental Information 8 [file peerj-14-20918-s008.pdf]
